# HIV-1 capsids from B27/B57^+^ elite controllers escape Mx2 but are targeted by TRIM5α, leading to the induction of an antiviral state

**DOI:** 10.1371/journal.ppat.1007398

**Published:** 2018-11-12

**Authors:** Natacha Merindol, Mohamed El-Far, Mohamed Sylla, Nasser Masroori, Caroline Dufour, Jia-xin Li, Pearl Cherry, Mélodie B. Plourde, Cécile Tremblay, Lionel Berthoux

**Affiliations:** 1 Laboratory of antiviral immunity, Department of medical biology, Université du Québec à Trois-Rivières, Trois-Rivières, Quebec, Canada; 2 Department of microbiology, infectiology and immunology, Centre de Recherche du Centre Hospitalier de l’Université de Montréal, Montréal, Canada; Fred Hutchinson Cancer Research Center, UNITED STATES

## Abstract

Elite controllers (ECs) are a rare subset of HIV-1 slow progressors characterized by prolonged viremia suppression. HLA alleles B27 and B57 promote the cytotoxic T lymphocyte (CTL)-mediated depletion of infected cells in ECs, leading to the emergence of escape mutations in the viral capsid (CA). Whether those mutations modulate CA detection by innate sensors and effectors is poorly known. Here, we investigated the targeting of CA from B27/B57^+^ individuals by cytosolic antiviral factors Mx2 and TRIM5α. Toward that aim, we constructed chimeric HIV-1 vectors using CA isolated from B27/B57^+^ or control subjects. HIV-1 vectors containing B27/B57^+^-specific CA had increased sensitivity to TRIM5α but not to Mx2. Following exposure to those vectors, cells showed increased resistance against both TRIM5α-sensitive and -insensitive HIV-1 strains. Induction of the antiviral state did not require productive infection by the TRIM5α-sensitive virus, as shown using chemically inactivated virions. Depletion experiments revealed that TAK1 and Ubc13 were essential to the TRIM5α-dependent antiviral state. Accordingly, induction of the antiviral state was accompanied by the activation of NF-κB and AP-1 in THP-1 cells. Secretion of IFN-I was involved in the antiviral state in THP-1 cells, as shown using a receptor blocking antibody. This work identifies innate activation pathways that are likely to play a role in the natural resistance to HIV-1 progression in ECs.

## Author summary

Some HIV-1-infected individuals show a natural capacity to control viral propagation. In individuals that have the HLA B27 or B57 allele, HIV-1 control is associated with mutations in viral proteins that arise as a result of immune pressure from cytotoxic T lymphocytes. HIV-1 capsid protein mutations found in these subjects render HIV-1 more sensitive to detection by TRIM5α, a cytoplasmic innate effector that targets retroviral capsids. We show here that HIV-1 bearing such mutations is restricted by TRIM5α but not by Mx2, another capsid-targeting innate effector. As a result, cells have decreased permissiveness to subsequent HIV-1 infections, a phenomenon that could contribute to the inefficient disease progression observed in these individuals. This knowledge might find applications in the development of immune interventions to increase human cells resistance to HIV-1.

## Introduction

ECs are a rare (<0.5%) and heterogeneous subset of HIV-1-infected subjects grouped together because they maintain undetectable viremia (<50 copies⁄ml) and normal CD4^+^ T cell counts in the absence of antiretroviral therapy (ART). Peripheral virus is usually not detectable by conventional PCR methods but low-level replication is ongoing [1]. While their viremia is controlled, these individuals present a persistent low-grade inflammation and, when compared to ART-treated individuals, they are at a higher risk of hospitalization due to chronic inflammation-related problems such as cardiovascular diseases [2, 3]. Genetic studies have shown that HLA alleles such as B57 and B27 contribute to the success of the CD8^+^ cytotoxic T lymphocyte (CTL)-mediated depletion of infected cells [4–6]. The retroviral CA protein is one of the most successful targets of the CTL response [7, 8], and this immune pressure drives the emergence of escape mutations in CA.

CA is also the target of innate immune restriction factors that act on the retroviral CA core following its release in the cytosol upon entry, including the interferon-stimulated genes (ISGs) Mx2/MxB (Myxovirus-resistance protein 2 or B) and TRIM5α (Tripartite motif-containing protein 5, isoform α) [9–11]. The dynamin-like GTPase Mx2 was first identified in 2013 as a key inhibitor of HIV-1 replication following type I interferon (IFN-I) treatment [12–14]. Mx2 inhibits viral core disassembly, impedes with viral genome nuclear import and possibly with post-nuclear entry steps [13, 15, 16]. The anti-HIV-1 activity of rhesus TRIM5α was described almost a decade earlier [17]. TRIM5α is part of a large family of proteins containing a tripartite motif [18]. At its C-terminus is a variable B30.2/SPRY domain that determines the specificity of the restriction, *i*.*e*. which viruses are targeted by a particular TRIM5α ortholog [19–21]. Recognition of an incoming retrovirus through interactions between TRIM5α and its specific CA target impairs the progression of the infection by several mechanisms including the accelerated disassembly of the retroviral CA core, accompanied by a decrease in the amount of reverse transcription (RT) products [22–24]. As a consequence, core components such as viral RNA and integrase are solubilized or degraded [25] (reviewed in [26]). Mx2 and TRIM5α both act in a cell type-, species- and viral strain-specific manner and the CA N-terminal domain is the main viral determinant of sensitivity to both restriction factors [15, 24, 27–31].

In addition to its effector functions, TRIM5α acts as a pattern recognition receptor (PRR), *i*.*e*. an innate sensor of the retroviral CA [32–35]. The TRIM5α N-terminal RING domain recruits the E2-ubiquitin conjugating enzyme heterodimer Ubc13 (Ube2N)/Uev1a (or Uev2) to generate lysine 63 (K63)-linked polyubiquitin chains [34, 35] that can be anchored onto TRIM5α through the action of another E2 enzyme, Ube2W [32, 33]. K63-linked ubiquitin, in association with the TAK1 kinase complex, leads to the activation of both NF-κB and AP-1 pro-inflammatory pathways [33, 34, 36, 37].

Early studies showed that in contrast to Mx2, the human ortholog of TRIM5α (huTRIM5α) does not significantly restrict laboratory strains of HIV-1 [17, 38]. However, a more recent study showed that CTL escape CA mutants found in EC subjects carrying the alleles HLA-B27 or B57 (B27/B57^+^) had increased sensitivity to restriction by huTRIM5α [39, 40]. This observation constituted the first evidence that huTRIM5α could target HIV-1, at least in these ECs. Consistently, genetic studies have repeatedly isolated polymorphisms in *TRIM5* modulating disease progression [9, 41–43]. However, whether endogenous TRIM5α can act as a PRR for HIV-1 CA in B27/B57^+^ ECs was not known. In addition, whether the CA mutants found in these subjects are more or less sensitive to Mx2 was not known either. We demonstrate here that in addition to blocking the replication of HIV-1 strains isolated from B27/B57^+^ subjects, endogenous huTRIM5α contributes to the induction of an antiviral state involving pro-inflammatory pathways, thereby shielding the cells against subsequent infections.

## Results

### HIV-1 CA variants isolated from B27/B57^+^ individuals exhibit a high number of CTL escape mutations, elude Mx2 restriction but are sensitive to TRIM5α

**Fig 1:**
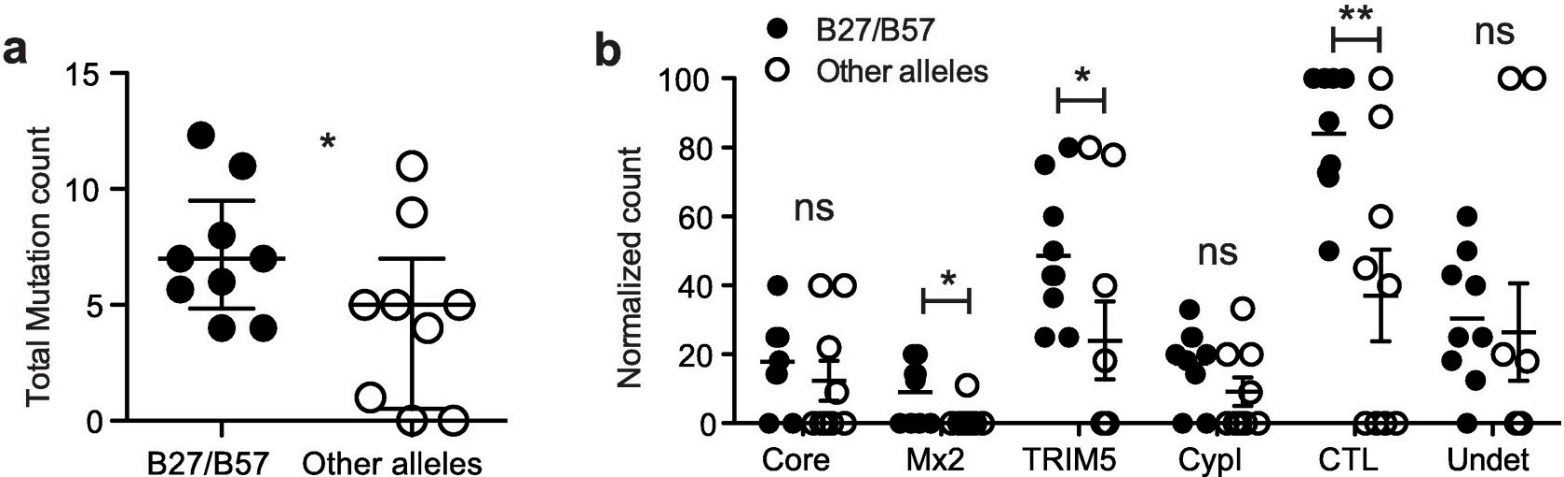
High frequency of mutations associated with sensitivity to huTRIM5α in CA from B27/B57^+^ individuals. **(a)** Polymorphisms in the aa regions 133–138, 146–173, 215–228, 238–250 and 262–273 of CA (displayed in S2 Table) were counted and grouped according to donor HLA alleles, *i*.*e*. B27/B57^+^ (NRC2, NRC10, EC1, EC3, EC5-9) or others (NRC1, NP1-6, NP8, NP10). For those subjects from whom two or more CA sequences were cloned (see S2 Table), the average number of mutations per subject was calculated. The Mann-Whitney test was used (*p* = 0.0499). **(b)** Polymorphisms in CA from subjects bearing B27/B57 alleles (n = 9) or not (n = 9) were counted according to their known association with decreased infectivity (Core), resistance to Mx2 (*p* = 0.0176, unpaired Student’s t-test), sensitivity to TRIM5α (*p* = 0.0335), resistance to CypA inhibitors (CypI), CTL escape/compensatory (CTL) (*p* = 0.0077) or of unknown function (undet). Data shown are normalized to the total number of mutations observed for each patient.

To define the restrictive potential of endogenous human Mx2 and TRIM5α against HIV-1 in B27/B57^+^ individuals, we included all 9 B27/B57^+^ subjects from the Canadian Slow Progressors cohort (S1 Fig), and we were able to amplify CA sequences from 7 of them (EC1, EC3, EC5-9). Of note, EC9 had reverted from the EC status at the time of sample collection (S1C Fig). Two additional isolates from B27/57 individuals (NRC2, NRC10) previously shown to be huTRIM5α-sensitive [39] were also included (S1 Table; S1 References). As a control group, sera were obtained from 10 non-B27/B57 normal progressors, and we were able to amplify CA sequences from 8 of them (NP1-6, NP8, NP10). The control virus NRC1 [39] and a laboratory strain (NL4-3) were also included in the study. CA sequences were amplified following RNA extraction from donors’ plasma and inserted into pNL4-3_GFP_ and pNL4-3_DsRed_ to generate chimeric vectors that were then sequenced (S2 Table; S1 References). Some plasma samples yielded two or more CA variants (S2 Table). The CA sequence of pNL4-3 (NY5) was used as reference. Polymorphisms in the IW9, KF11, TW10 and KK10 epitopes and the CypA-binding loop of the N-terminal region previously associated with immune pressure were tallied [39, 44, 45] (S2 Table, Fig 1A). A mean of 7.2 mutations were found in viruses isolated from B27/B57^+^ subjects (n = 9) compared to 4.4 in viruses derived from subjects having other alleles (n = 9) (*p =* 0.049). To further analyze differences in the whole CA sequence between the two groups of subjects, for each subject we enumerated the mutations previously associated with: i) decreased core stability, ii) escape from Mx2 restriction, iii) increased sensitivity to TRIM5α restriction, iv) increased resistance to cyclophilin A inhibitors (CypI), v) CTL escape or compensation to CTL escape, and vi) unknown function (Fig 1B; S3 Table; S1 References). Mutations known to modulate core stability and CypI resistance were found at a similar frequency in the two groups. CTL escape/compensatory mutations were more frequently detected in viruses derived from B27/B57^+^ vs other subjects (*p* = 0.0077). Mutations induced by CTL pressure were often associated with several other phenotypes. For instance, G116A was described to both increase sensitivity to TRIM5α and decrease restriction by Mx2 [39, 46], while R132K was linked with decreased core stability, increased CypI resistance and increased sensitivity to TRIM5α [46] (S3 Table). Globally, there was an increase in the Mx2 resistance G116A polymorphism in the B27/B57 group of subjects (*p* = 0.0176; Fig 1B). Other polymorphisms at the G116 position (*i*.*e*. 116R and 116Q) were observed but no other mutation previously reported to confer resistance to Mx2 was found. Interestingly, we detected about twice as many polymorphisms potentially conferring sensitivity to huTRIM5α in B27/B57^+^ subjects, relative to subjects bearing other alleles (*p* = 0.0335). Overall, sequence analyses suggest that CTL escape mutations may affect sensitivity to CA-targeting restriction factors. Using the Chi-square and Fisher’s exact tests, we could confirm that aminoacid 116 was more frequently mutated in viruses from B27/B57 subjects, and that the latter were also more likely to have viruses with at least one mutation altering the sensitivity to TRIM5α (S2A and S2B Fig).

#### HIV-1 strains found in B27/57+ individuals are sensitive to restriction by TRIM5α

**Fig 2:**
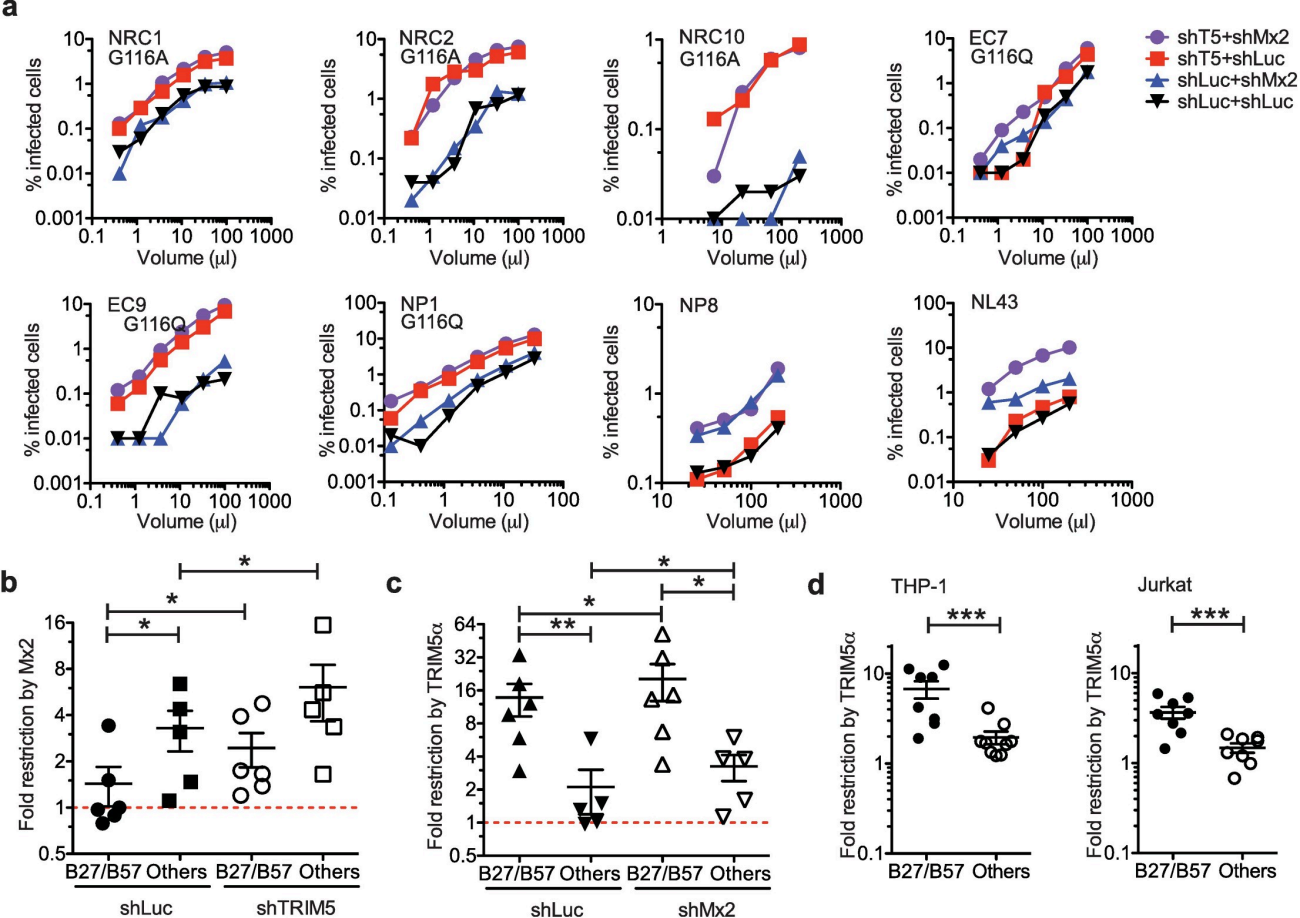
CA isolated from B27/B57^+^ HIV-1^+^ individuals escape Mx2 restriction but display high sensitivity to TRIM5α. **(a)** THP-1 cells knocked down for TRIM5α (shT5) or Mx2 (shMx2) or both were treated with IFN-β and then infected with the indicated volumes of 7 representative chimeric vector preparations bearing CA from clinical isolates or from NL4-3. shLuc is used as a knockdown negative control. Mutations at G116 are specified for each vector. **(b)** The -fold restriction by Mx2 in THP-1 cells was quantified for chimeric HIV-1 vectors bearing CA isolated from B27/B57^+^ (EC5, EC7-9, NRC2, NRC10) and control subjects (NP1, NP4, NP8, NRC1, NL43). Restriction was assessed in TRIM5α-depleted cells (shTRIM5α) and in control cells (shLuc). Restriction by Mx2 was significantly lower for vectors bearing CA from B27/B57^+^ compared to controls (*p* = 0.0411; Mann-Whitney test), and significantly higher in TRIM5α knockdown compared to control cells (*p* = 0.0156 in B27/B57^+^ and *p* = 0.0313 in others; Wilcoxon's matched-pairs signed rank test). **(c)** Restriction of the chimeric vectors by TRIM5α in THP-1 cells was quantified in Mx2-depleted and control cells. Restriction by TRIM5α was significantly higher for vectors bearing CA from B27/B57^+^ compared to other subjects in both shLuc and shMx2 cells (*p* = 0.0043 and *p* = 0.0152, Mann-Whitney test). TRIM5α-mediated restriction was significantly higher in Mx2 knockdown compared to control cells for both B27/B57^+^ and other subjects (*p* = 0.0156 in B27/B57^+^ and *p* = 0.0313 in others; Wilcoxon's matched-pairs signed rank test). **(d)** TRIM5 knockout and control cells were infected with vectors bearing CA from B27/B57^+^ (EC3, EC5-9, NRC2, NRC10) or from other donors (NP1-4, NP6, NP8, NP10, NRC1 and the NL43 control) and the -fold restrictions were quantified for THP-1 cells (*p* = 0.0005) and Jurkat cells (*p* = 0.001). For subjects from which more than one CA sequences were recovered, we determined the -fold restriction for each (S2 Table) and the mean value was used here. Shown are means with SEM, and medians with full range in box and whiskers graphs. The unpaired Student’s *t*-test was used.

To evaluate the relative impacts of Mx2 and TRIM5α on viral replication, we knocked them down in monocytic THP-1 and lymphoid Jurkat cells (S3 Fig) and measured infectious titers of the NL4-3-based chimeric HIV-1 vectors harbouring CA originating from B27/B57^+^ subjects or from control subjects, following IFN-β treatment (Fig 2A, S4 Fig). To our surprise, for most viruses with the exception of EC7, NP8 and NL4-3, restriction by huTRIM5α seemed to be stronger than the one mediated by Mx2. This suggested that mutations G116A, G116R and G116Q found in the B27/B57^+^ isolates tested greatly reduced sensitivity to Mx2. Interestingly, knocking down both restriction factors significantly increased NL4-3 infectivity compared to the single knockdown of either restriction factor, but this effect was not observed with other viruses (Fig 2A, S4 Fig). Fold-restriction scores were calculated as the ratio between viral vector titers in the control cells divided by titers in knocked down cells in titration experiments. This analysis showed that chimeric vectors with the CA derived from randomly chosen B27/B57^+^ subjects (n = 6) were less sensitive to Mx2 than viruses from other subjects (n = 5) (*p* = 0.0411; means of 1.43 *vs*. 3.30, respectively) (Fig 2B). Mx2 sensitivity of B27/B57^+^ vectors was slightly increased upon TRIM5α knockdown (*p* = 0.0156 in B27/B57^+^ and *p* = 0.0313 in others). These results suggest that Mx2 does not target the CA from B27/B57^+^ subjects included in this study. By contrast, restriction by TRIM5α was more efficient on CA from B27/B57^+^ subjects compared to control subjects (*p =* 0.0043; means of 13.79 *vs*. 2.11, respectively) and was increased upon Mx2 knockdown (*p* = 0.0156 in B27/B57^+^ and *p* = 0.0313 in others) (Fig 2C).

To further analyze sensitivity to TRIM5α, *TRIM5* was knocked out in THP-1 and Jurkat cells using the CRISPR-Cas9 nuclease system with a TRIM5-targeting guide RNA (gRNA) (S5A–S5F Fig, S5 Table) [47]. As a negative control, a gRNA targeting a nonhuman sequence (“CAG”) was used. Infection of IFN-β-treated *TRIM5* knockout (T5KO) and control cells with the chimeric vectors allowed us to determine TRIM5α-dependent restriction levels. The mean -fold restrictions varied from 1.5 to 3.7 in Jurkat cells and from 2.0 to 6.8 in THP-1 cells infected with non-B27/B57-derived and with B27/B57-derived vectors, respectively (*p* = 0.001 in Jurkat and *p* = 0.0005 in THP-1) (Fig 2D). Thus, the chimeric vectors with B27/B57-derived CA were significantly more restricted by TRIM5α in both cell lines.

### TRIM5α activation of pro-inflammatory signals triggers an antiviral state that shields cells against further infections

**Fig 3:**
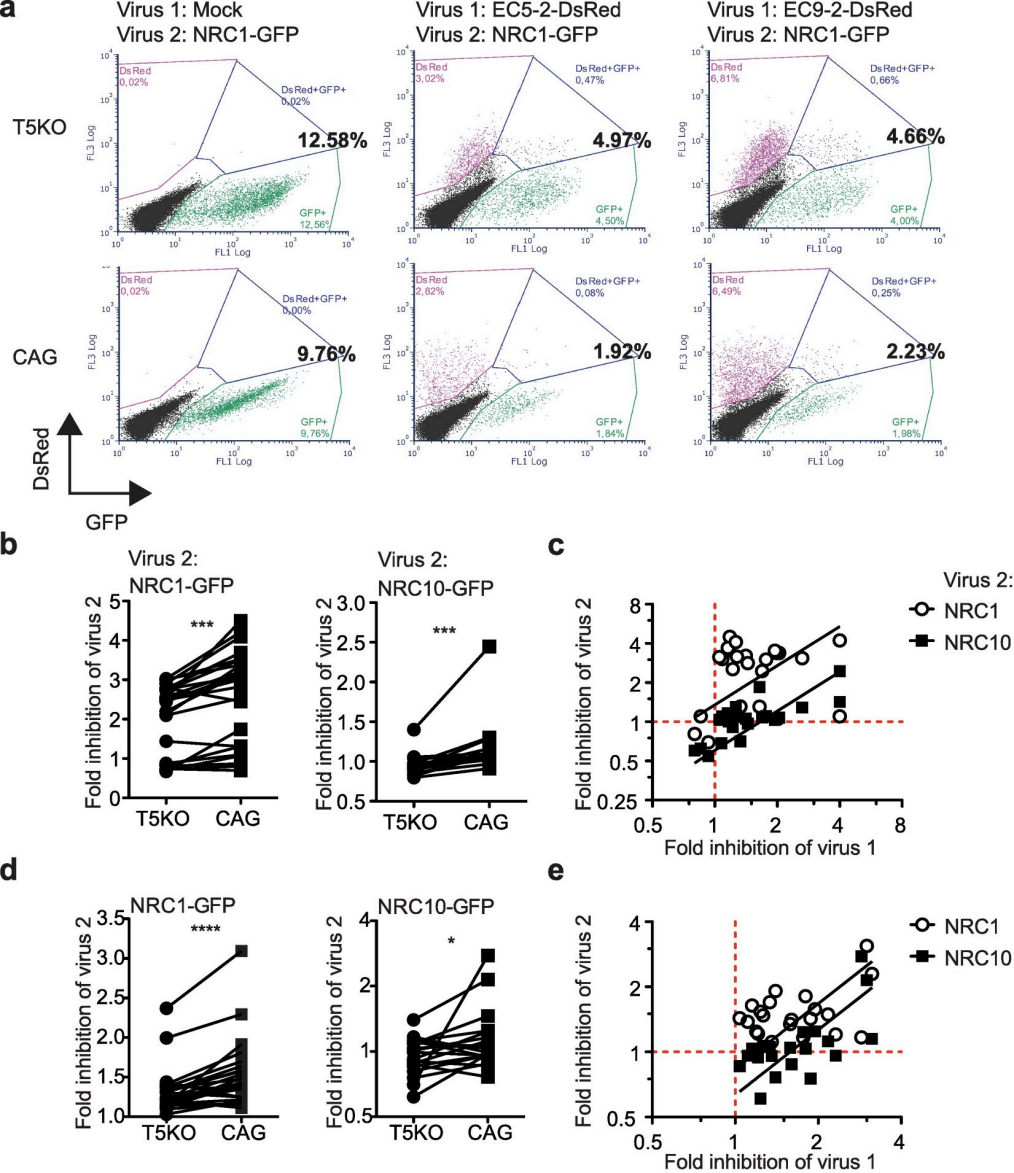
TRIM5α induces an antiviral state following infection with restricted viruses. **(a)** Representative two-color dot plots of T5KO and control (CAG) Jurkat cells infected first with B27/B57^+^ chimeric HIV-1 vectors EC5-2_DsRed_ or EC9-2_DsRed_, or mock-infected, followed 48 h later by a second infection with NRC1_GFP_. The total percentage of GFP^+^ cells is shown in bold. **(b)** Fold decreases in NRC1_GFP_ (n = 25) and NRC10_GFP_ (n = 17) infectivity in T5KO and control (CAG) Jurkat cells subjected to a first infection with isolates-derived DsRed-vectors. The Wilcoxon's matched pair signed rank test was used. **(c)** Correlation between the magnitude of TRIM5α-dependent inhibition of isolate-chimeric DsRed-expressing vectors (virus 1) and the strength of inhibition of virus 2 (NRC1_GFP_ or NRC10_GFP_) analyzed by linear regression (*p*<0.0001 for both viruses, n = 24) and Spearman’s non-parametric correlation in control (CAG) Jurkat cells (NRC1, *p =* 0.0690; NRC10, *p =* 0.0003). **(d)** Fold decrease of NRC1_GFP_ and NRC10_GFP_ infectivity following exposure to isolate-derived DsRed-expressing HIV-1 vectors in TRIM5-KO (T5KO) and control (CAG) THP-1 cells (*p*<0.0001 for NRC1 and *p* = 0.0411 for NRC10; n = 21). The Wilcoxon's matched pair signed rank tests were used. **(e)** Correlation between the magnitude of TRIM5α-dependent inhibition of isolate-chimeric vectors (“virus 1”) and the strength of inhibition of NRC1_GFP_ or NRC10_GFP_ (“virus 2”) analyzed by linear regression (p<0.0001 for both viruses, n = 21) and Spearman’s non-parametric correlation in control (CAG) THP-1 cells (NRC1_GFP_, *p =* 0.2340, r = 0.1675; NRC10_GFP_, *p =* 0.0054, r = 0.5443).

Pro-inflammatory and IFN-I signaling induce an antiviral state against HIV-1 in human cells [48, 49], but the possibility that TRIM5α-mediated restriction of HIV-1 contributes to inducing the antiviral state has never been explored. We set up an assay to quantitate this antiviral state by performing two infections with HIV-1 vectors, 48 h apart. We used vectors carrying two different fluorescent markers to analyze the cells’ permissiveness to each vector simultaneously by flow cytometry, as shown in Fig 3A. We observed in both THP-1 and Jurkat cells that pre-infection with a TRIM5α-sensitive HIV-1 vector resulted in a significant decrease in infectivity of the second virus (*i*.*e*. an antiviral state), regardless of whether this second vector was TRIM5α-sensitive (NRC10; *p =* 0.0002; n = 17) or -resistant (NRC1; *p*<0.0001; n = 25) (Fig 3A, 3B and 3D). In the representative FACS dot plots shown in Fig 3A, pre-infection with EC5-2_DsRed_ or EC9-2_DsRed_ resulted in a 5.1- and 4.4-fold decrease in infectivity for the second virus, respectively. In the *TRIM5* knockout cells, the reduction in infectivity of the second virus was smaller (2.5-fold and 2.7-fold). In addition, the strength of the antiviral state was significantly linked to the intensity of inhibition of the first virus by TRIM5α as determined by linear regression (*p*<0.0001 for NRC1 and NRC10 as second virus in THP-1 and Jurkat) (Fig 3C, n = 24; Fig 3E, n = 21) or by direct Spearman correlation (*p =* 0.0003, r = 0.6497 in Jurkat and *p* = 0.0054, r = 0.5443 in THP-1) (Fig 3C, Fig 3E). Altogether, these results strongly suggest that upon infection with a restriction-sensitive HIV-1, huTRIM5α induces an antiviral state resulting in the inhibition of viruses regardless of their sensitivity to huTRIM5α.

### The TRIM5α-mediated antiviral state does not require a productive infection

**Fig 4:**
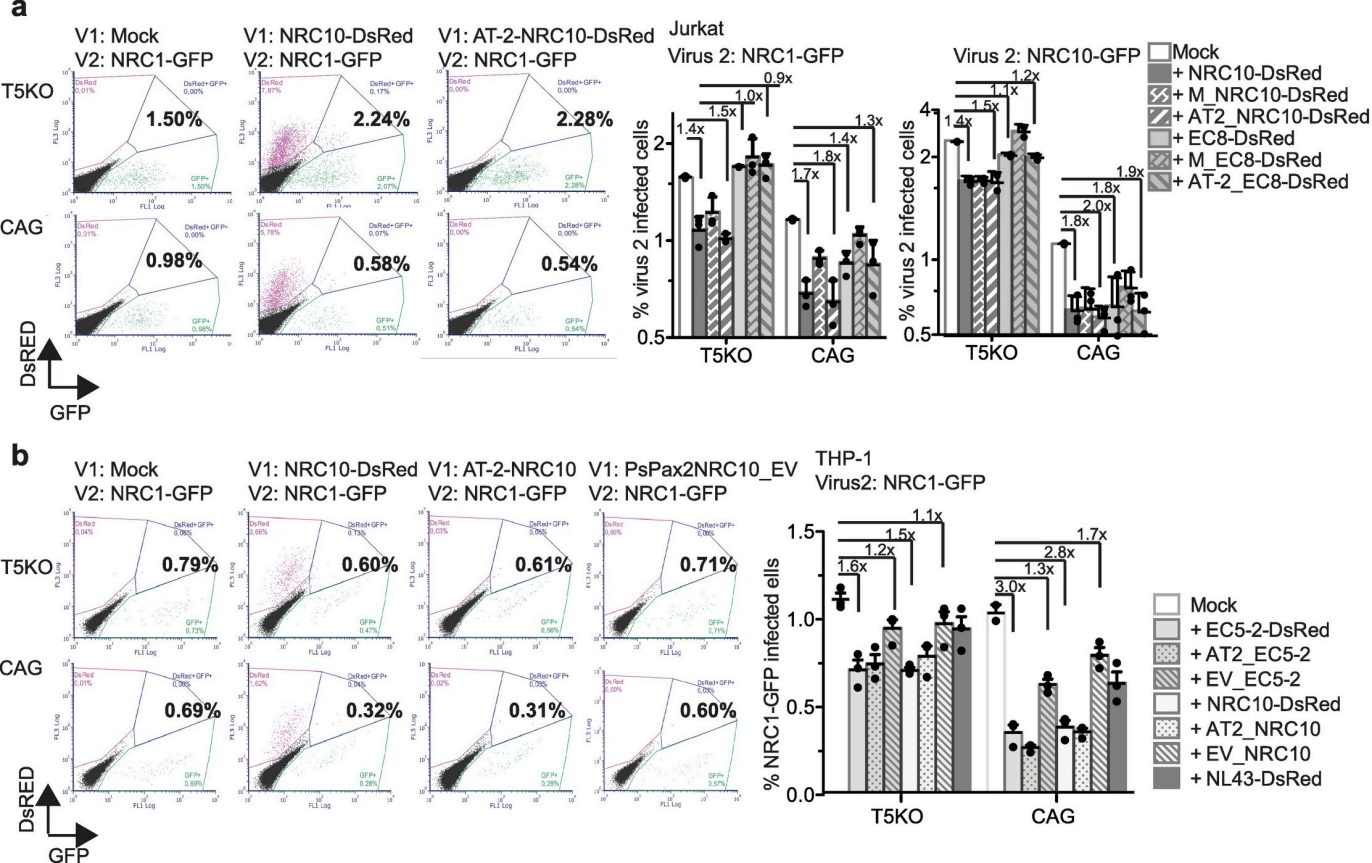
Induction of the TRIM5α-activated antiviral state does not require a productive infection. **(a)** Representative FACS dot plots of TRIM5-KO or control Jurkat cells infected or not with AT-2- or MetOH (vehicle)-treated NRC10_DsRed_, followed by challenge with NRC1_GFP_ 48 h later. The % of GFP^+^ cells are shown in bold. Bar graphs showing the percentage of infected cells following infection of T5KO or control Jurkat cells with NRC1_GFP_ or NRC10_GFP,_ 48 h after a first infection with NRC10_DsRed_ or EC8-2_DsRed_ treated with AT-2 or with the vehicle only (M). Means with SD are plotted. Numbers on the bars represent the -fold inhibition relative to the relevant mock control. **(b)** Representative dot plots of TRIM5-KO and control THP-1 cells infected or not with AT-2- or vehicle-treated NRC10_DsRed_, or with “empty” psPAX2-vector containing NRC10 CA (NRC10_EV), followed by NRC1_GFP_ challenge 48 h later. The % of GFP^+^ cells are shown in bold. Bar graphs showing the percentage of infected cells following infection of T5KO or control THP-1 cells with NRC1_GFP,_ 48 h after a first infection using NRC10_DsRed_ or EC5_DsRed_ treated with AT-2, with the vehicle only, or using NRC10 or EC5-2 “empty vectors”. Means with SD are plotted. Numbers on the bars represent the -fold inhibition relative to the relevant mock control. Shown in all bar graphs of this figure are representative experiments performed in triplicates and repeated at least three times. Individual values are included (black symbols).

Next, we investigated whether the interaction between TRIM5α and the CA lattice was sufficient to trigger an antiviral state. For this, we inactivated HIV-1 vector particles using AT-2 (Aldrithiol-2), a compound that covalently modifies the nucleocapsid protein zinc fingers and therefore abrogates productive infection, while maintaining the conformational integrity of the viral envelope and capsid [50]. Thus, TRIM5α-CA interactions are expected to occur but the viral life cycle is stopped pre-completion of reverse transcription. We infected TRIM5 knockout and control Jurkat and THP-1 cells with TRIM5α-sensitive vectors (NRC10, EC8, EC5-2) that were treated or not with AT-2, and could not detect any productive infection upon inactivation of the vectors with AT-2, as expected (Fig 4A and 4B). 48 h later, cells were infected with the NRC1_GFP_ (TRIM5α-insensitive) or NRC10_GFP_ (TRIM5α-sensitive) vectors. In the control cells expressing TRIM5α, we consistently observed an antiviral state inhibiting the second virus by ~1.5- to 3-fold, whether NRC1_GFP_ or NRC10_GFP_ was used (Fig 4A and 4B). In all cases, we observed no significant difference between the antiviral state induced by untreated and AT-2-treated vectors, indicating that induction of an antiviral state does not require completion of reverse transcription nor subsequent steps. We also constructed psPAX2-based “empty” viral (EV) chimeric particles bearing the CA from the TRIM5α-sensitive EC5-2 and NRC10 that do not contain viral RNA. The amounts of regular and EV vectors used were equalized by reverse transcriptase activity. THP-1 cells were infected with NRC10_EV or EC5-2_EV, and then infected with NRC1-GFP 48h later (Fig 4B). We observed that the antiviral state (*i*.*e*. the inhibition of NRC1-GFP) was modest when NRC10_EV or EC5-2_EV was used as the first virus, similar to the TRIM5α-insensitive NL4-3-DsRed (Fig 4B). This result suggests that efficient induction of the antiviral state requires the presence of a factor that is absent from the psPAX2-based EVs.

### The antiviral state is associated with upregulated innate signaling pathways

**Fig 5:**
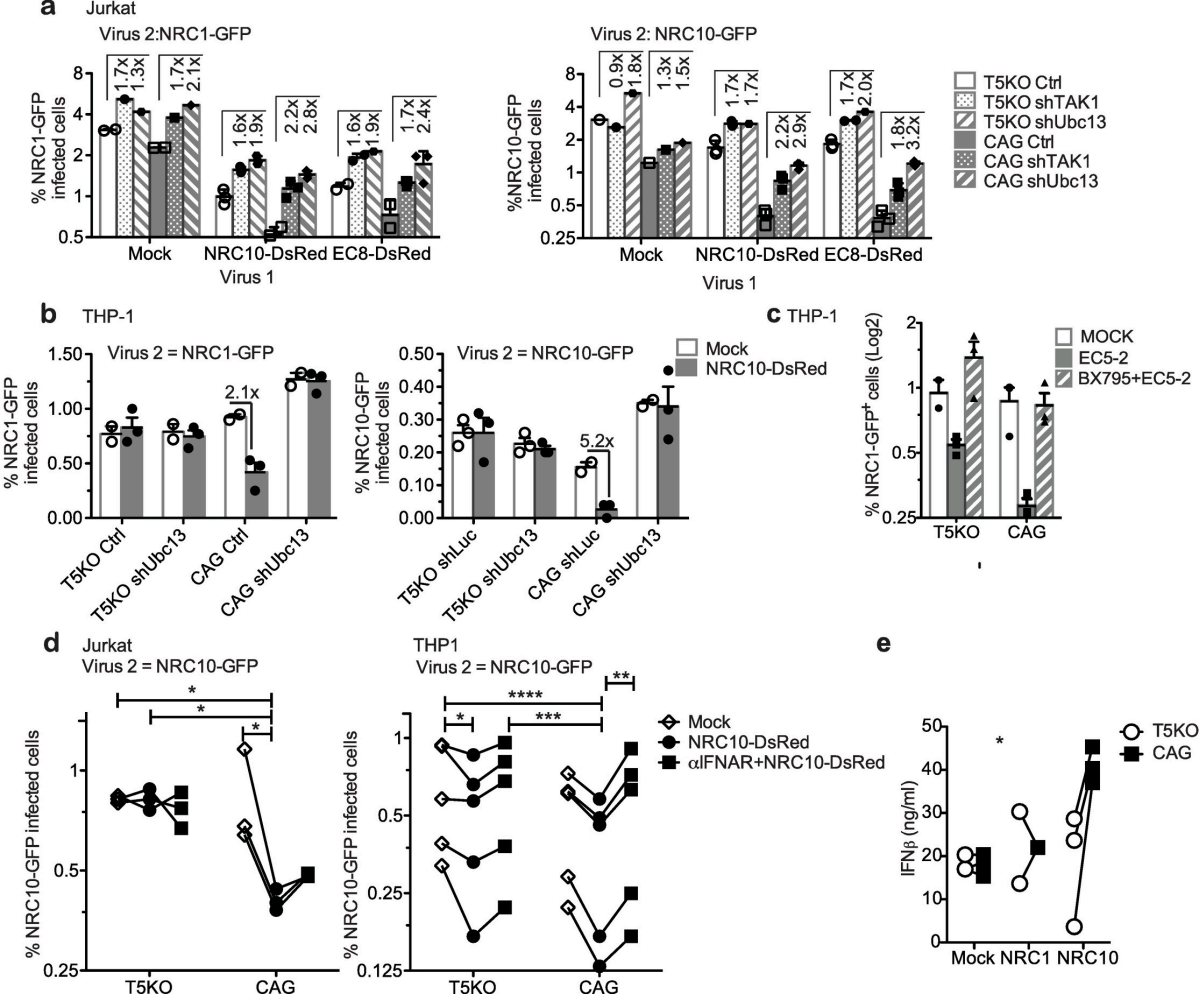
The TRIM5α-induced antiviral state depends on pro-inflammatory pathways. **(a)** T5KO or control Jurkat cells transduced with shRNAs targeting TAK1 or Ubc13 or transduced with a control shRNA were infected with NRC10_DsRed_ or EC8_DsRed_ and 48 h later with “virus 2” NRC1_GFP_ (left panel) or NRC10_GFP_ (right panel). Virus 2 infectivity was measured by FACS two days later. Fold increases in the percentage of virus 2-infected cells in shTAK1 and shUbc13 cells relative to the control cells are shown. Pre-infection with virus 1, TRIM5 knockout, and Ubc13 or TAK1 knockdown significantly impacted the infectivity of both NRC1_GFP_ and NRC10_GFP_ (p<0.0001, two-way ANOVA). **(b)** T5KO or control THP-1 cells were depleted of Ubc13 (shUbc13) or not. Cells were then infected with NRC10_DsRed_ vectors (“virus 1”), and subsequently with NRC1_GFP_ (left panel) or NRC10_GFP_ (right panel). Bars are means with SEM. Experiments were performed three times in triplicates. TRIM5 knockout and Ubc13 knockdown significantly impacted the infectivity of both NRC1_GFP_ and NRC10_GFP_ (*p* = 0.0368 and *p* = 0.0035, respectively) (two-way ANOVA). **(c)** Infectivity levels of virus 2 (NRC1_GFP_) in TRIM5-KO and control THP-1 cells pre-treated or not with the TBK1/IKKε inhibitor BX795 for 1 h at 5 μM before the infection with EC5-2-DsRed. **(d)** IFNα/βR2 was neutralized or not by pre-treatment of Jurkat and THP-1 cells with a monoclonal antibody. Cells were then infected with NRC10_DsRed_ or not and 48 h later with NRC10_GFP_. The % of GFP^+^ cells was measured by FACS. The One-way ANOVA with repeated measures and the Bonferroni’s multiple comparison tests were used (*p<0.05, **p<0.001, ***p<0.0001; Jurkat, n = 3; THP-1, n = 5). **(e)** IFN-β levels in supernatants of TRIM5-KO and control THP-1 cells infected or not with the NRC1_DsRed_ and NRC10_DsRed_ vectors, measured by ELISA and compared using a 2-way ANOVA.

TRIM5α can activate innate immune pathways through its E3 ubiquitin ligase activity that cooperates with the E2 ligase complex Ubc13/UEV2A to generate K63-linked ubiquitin chains, which in turn activate TAK1, the kinase that phosphorylates the IKK complex as well as the IKK-related kinases TBK1 and IKKε [51, 52]. IKK and related proteins phosphorylate the NF-κB inhibitor IκB, leading to NF-κB activation [53, 54]. TAK1 also mediates the activation of AP-1 through a different mechanism [55]. Thus, TRIM5α stimulates pro-inflammatory pathways leading to the activation of NF-κB and AP-1, which may result in IFN-I secretion [33, 34, 37, 56]. We tested whether these downstream mediators of TRIM5α-dependent signaling had a role in the antiviral state. Depletion of TAK1 and Ubc13 (S5G Fig) resulted in an attenuated antiviral state both in Jurkat and THP-1 cells (Fig 5A and 5B). In addition, the TBK1/IKKε signaling inhibitor BX795, which targets TBK1 [57], abrogated the TRIM5α-dependent induction of an antiviral state (Fig 5C; see effect on virus 1 in S6A Fig). These results indicate that the antiviral state dependent on huTRIM5α is mediated by the signal transducers Ubc13, TAK1 and TBK1. In order to evaluate the importance of IFN-I signaling in the induction of the antiviral state, cells were treated with an antibody against IFNα/βR2 1 h prior to infection with the first virus (NRC10_DsRed_) (Fig 5D). In Jurkat cells expressing TRIM5α, a strong antiviral state was induced following infection with a TRIM5α-sensitive virus (NRC10), which was slightly reduced but not abrogated upon neutralization of the type I IFN receptor. By contrast, treatment with the neutralizing antibody completely prevented the induction of a antiviral state in THP-1 cells (Fig 5D). Consistently, there were significantly higher levels of IFN-β in the supernatants of THP-1 cells expressing TRIM5α and infected with a TRIM5α-sensitive virus than in non-infected cells or in cells that did not express TRIM5α (*p =* 0.0270, effect of TRIM5α by 2-way ANOVA; Fig 5E). Using the same ELISA test, we could not measure any detectable levels in Jurkat cells. These results highlight the role of type I IFN in the establishment of the antiviral state in THP-1 cells but not in Jurkat cells.

#### NF-κB and AP-1 are involved in the antiviral state in THP-1 cells

**Fig 6:**
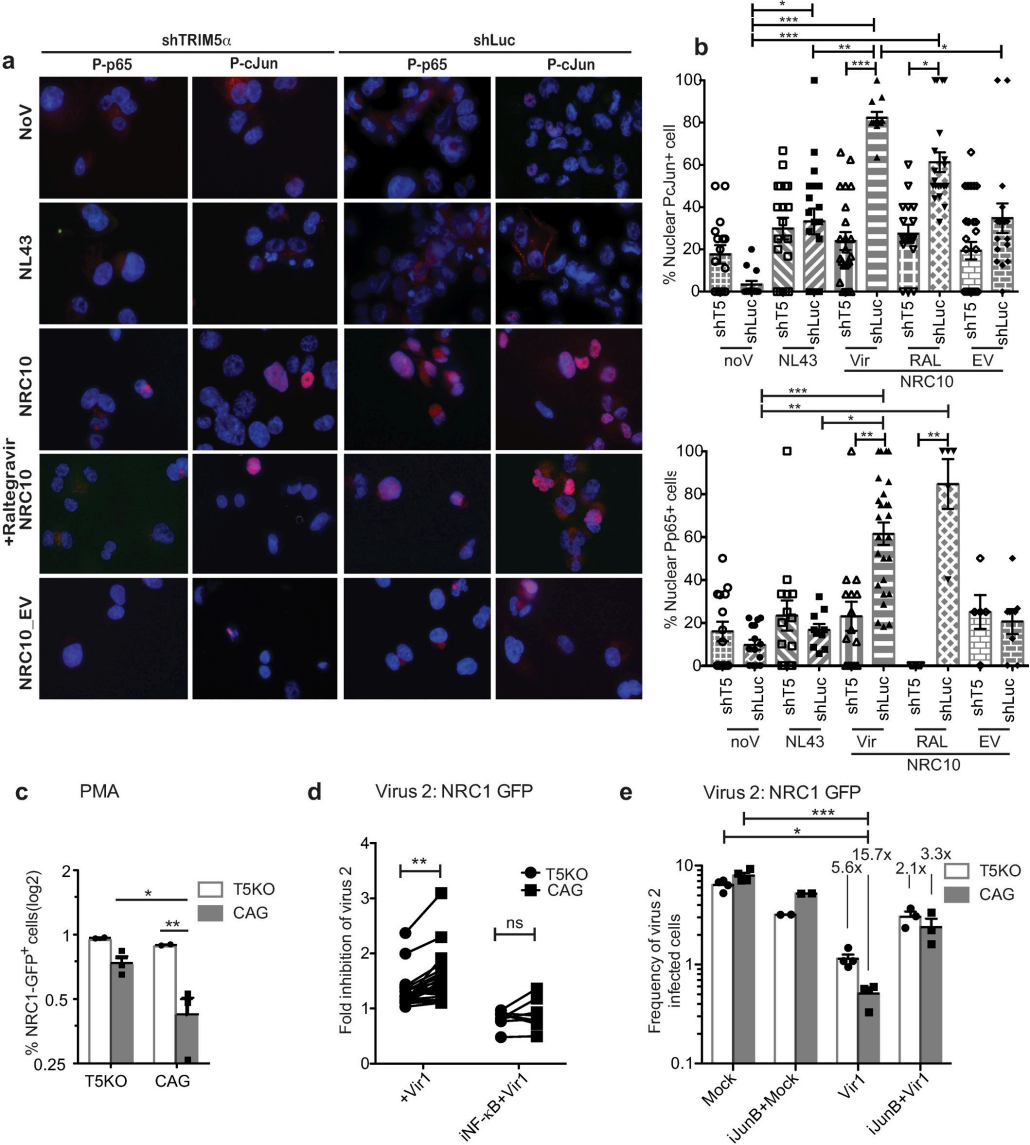
NF-κB and AP-1 are involved in the induction of the antiviral state in THP-1 cells. **(a)** Representative IF microscopy staining of P-p65 and P-cJun in shTRIM5 and control shLuc PMA-differentiated THP-1 cells following infection for 48 h with NL43_GFP_ and NRC10_GFP_ (CRFK MOI = 10), as well as following infection with empty vectors. Where indicated, cells were pre-treated with Raltegravir for 1h. **(b)** Frequency of nuclear P-cJun and P-p65 quantified by analyzing ≥100 cells from ≥ 10 pictures and plotted according to TRIM5α expression and viral infection. The Kruskal-Wallis test and the Dunn's Multiple Comparison Test were used to assess statistical significance. Shown are means with SEM. noV = No virus, Vir = virus, RAL = Raltegravir, EV = empty vector. **(c)** Infectivity levels of virus 2 (NRC1_GFP_) in TRIM5-KO and control PMA-differentiated THP-1 cells pre-infected with NRC10_Ds-Red_. Differences in the % of GFP^+^ cells were analyzed using a two-way ANOVA. (**d**) THP-1 cells were treated with BAY11-7085 (“iNF-κB”) for 1 h and washed prior to infection with the patient-derived chimeric DsRed-expressing HIV-1 vectors (“virus 1”); 48 h later, they were exposed to NRC1_GFP_ (“virus 2”). The fold inhibition of NRC1_GFP_ infectivity was measured with or without inhibitor in T5KO and in CAG cells (*p =* 0.0085 in absence of inhibitor, n = 24; *p =* 0.2734 in presence of inhibitor, n = 8). The Wilcoxon's matched pair signed rank test was used. **(e)** THP-1 cells were pre-treated or not with SP600125 (“iJunB”) and washed before being infected with NRC10_DsRed_. 48 h later, cells were infected with NRC1_GFP_ and the % GFP^+^ cells were analyzed by FACS. Bars are means +SEM, and individual values are shown as well. Experiments were performed three times in triplicates. Numbers represent the fold decrease in % GFP^+^ cells relative to mock-infected cells treated similarly. The Kruskal-Wallis and Dunn’s multiple comparison tests were used (*p<0.05, ***p<0.0001).

To determine whether the transcription factors NF-κB and AP-1 were activated upon restriction of B27/57^+^ HIV-1 by huTRIM5α, we first analyzed the nuclear accumulation of phosphorylated NF-κB subunit p65 and phosphorylated AP-1 subunit cJun. THP-1 cells were differentiated into macrophage-like cells using PMA treatment for 24 h [58], then washed and rested for 72 h before being infected with NRC1_GFP_ or NRC10_GFP_ vectors for 48 h. Adherent THP-1 cells were then stained to gauge the nuclear translocation of phosphorylated p65 and cJun (Fig 6A; images including GFP fluorescence are shown in S7A Fig). We observed a strong activation of NF-κB and AP-1 in cells expressing TRIM5α and infected with the TRIM5α-sensitive NRC10 vector (quantifications are shown in Fig 6B). On the other hand, activation did not occur in cells knocked out for TRIM5α or infected with the TRIM5α-insensitive NL4-3. Blocking integration with Raltegravir abrogated infectivity of the HIV-1 vector used (S7B and S7C Fig) but had no significant effect on the activation of NF-κB and AP-1 (Fig 6A and 6B). Finally, infection with empty NRC10 vectors did not lead to significant NF-κB or AP-1 activation in the presence of TRIM5α (Fig 6A and 6B). Thus, infection conditions leading to an antiviral state correlated with the activation of NF-κB and AP-1 in this assay. Importantly, PMA-induced differentiation did not alter the TRIM5α-induced antiviral state in THP-1 cells (Fig 6C).

To investigate more directly the involvement of NF-κB in the antiviral state, we pre-treated THP-1 cells with its pharmacological inhibitor BAY11-7085. Treatment with the inhibitor for 1 h followed by washes prior to the first infection had little effect on the infectivity of the first virus (S6B Fig), whereas it prevented the induction of an antiviral state as we found no difference in the fold-inhibition of the second virus (NRC1_GFP_) between knockout and control cells following treatment (medians of 1.23-fold vs 1.43-fold in T5KO and control cells without treatment, *p =* 0.0085, n = 25; 0.85- *vs* 0.84-fold following treatment, *p =* 0.2734, n = 8; Fig 6D). We also pre-treated the cells with the cJun N-terminal kinase inhibitor SP600125 (Fig 6E) [33]. This AP-1 inhibitor generally increased HIV-1 infectivity (S6C Fig) but specifically decreased the induction of a TRIM5α-dependent antiviral state against virus 2 (NRC1_GFP_), as evidenced by the loss of virus 2 inhibition in CAG cells pre-infected with NRC10_DsRed_ (Fig 6E). Therefore, infection of THP-1 cells with TRIM5α-sensitive HIV-1 from B27/57^+^ individuals promotes the TRIM5α-dependent activation of NF-κB and AP-1.

## Discussion

The viral CA core stands under severe conformational constraints to remain functional [59] and is subjected to immune pressures from several sources, as it is targeted by both innate and adaptive immunity, *e*.*g*. restriction factors and CTLs. Here, we characterized the roles of Mx2 and TRIM5α in the successful control of HIV-1 that takes place in B27/B57^+^ individuals. First, we searched for footprints of CTL pressure and of modulation of sensitivity to Mx2 and TRIM5α in the CA sequences. Previous reports uncovered numerous mutation sites associated with escape from Mx2 restriction *in vitro* [27, 46, 60]. In our isolates, only one previously described Mx2 escape polymorphism was detected: G116A, a CTL escape mutation in the TW10 epitope that was more frequently detected in B27/B57^+^ subjects. We detected two other variants at this site, 116R and 116Q in 3 B27/B57^+^ subjects and 1 control that probably conferred resistance to Mx2 restriction as well. An intriguing point arising from our results is the overall low levels of restriction by Mx2 for most isolates. Natural evolution towards Mx2 escape has been reported previously in clinical isolates and was associated with the polymorphism 116A in HIV-1 Subtype C of Chinese origin [46]. In our analysis, restriction of CA isolated from B27/B57^+^ individuals was mostly undetectable, suggesting that this restriction factor does not participate in the control of HIV-1 in ECs. We cannot exclude, however, that incomplete depletion of Mx2 may have resulted in underestimating its restriction potency in these knockdown experiments. In contrast to Mx2, we observed an increase in huTRIM5α sensitivity for CA from B27/B57^+^ subjects. Interestingly, the strength of restriction by either TRIM5α or Mx2 doubled in absence of the other restriction factor. This suggests the existence of a negative competition effect between the two CA-binding factors whereby each disturbs the other one’s function.

A TRIM5α-dependent antiviral state was induced following infection with restriction-sensitive HIV-1 capsids. This antiviral state was reversed by depletion of the pro-inflammatory mediators TAK1 and Ubc13. In THP-1 cells, the TRIM5α-dependent antiviral state was associated with NF-κB and AP-1 activation, and inhibiting these transcription factors reduced the antiviral state. IFN-I receptor blockade prevented the antiviral state in THP-1 but less so in Jurkat cells, consistent with the absence of significant IFN-β production in Jurkat cells. Thus, the antiviral state is associated with activation of pro-inflammatory pathways that were previously shown to be triggered by TRIM5α in host-virus mismatch-species contexts [33, 34]. It is unclear whether the observed signaling strictly stems from CA-TRIM5α interactions, or whether TRIM5α might upregulate pro-inflammatory signaling stemming from other sensing events. AT-2 and Raltegravir treatments showed that the establishment of an antiviral state is independent of viral life cycle steps starting with reverse transcription. However, “empty” HIV-1 vectors devoid of viral RNA were less competent for the induction of the antiviral state, suggesting a possible role for viral RNA in this process. Interestingly, similar “empty” vectors based on N-MLV did activate the transcription of innate immunity-specific genes, probably in a NF-κB- and AP-1-dependent fashion, in the Pertel *et al* study [33]. We attribute this discrepancy to the much higher levels of N-MLV restriction by huTRIM5α (~100-fold, typically). Finally, both TRIM5α-sensitive and -resistant viruses were sensitive to the TRIM5α-dependent antiviral state, implying that unidentified, interferon-inducible effectors are involved (see theoretical model in S8 Fig).

In conclusion, this study shows that CTL escape mutants circumvent the restriction mediated by Mx2 but become more sensitive to the restriction factor TRIM5α. In addition to restricting the replication of sensitive HIV-1 strains found in B27/57^+^ individuals, TRIM5α induces an antiviral state in which permissiveness to subsequent HIV-1 infections, including with TRIM5α-insensitive viruses, is decreased. Future experiments will need to characterize this antiviral pathway in primary cells and to identify the effectors of the antiviral state. Replication-incompetent virus-like particles able to be sensed by and activate endogenous human TRIM5α may constitute the basis for the development of novel approaches aimed at decreasing permissiveness toward HIV-1.

## Materials and methods

### Ethics statement

This study (SL-04-061) was approved by the Institutional Review Boards at all participating sites. All patients were enrolled in the study following written informed consent. The study involved no animals. The ethics certificates are as follows: Centre Hospitalier de l’Université de Sherbrooke, 10–015; McGill University Medical Center, GEN-05-13, GEN-04-039; Centre Hospitalier de l’Université de Montréal, SLA04,061; Centre Hospitalier Universitaire de Québec, 2012–432 CH09-08-080; Clinique Quartier Latin and Clinique Médicale l’Actuel, 5005–10:37:1824-04-2017; Ottawa Hospital Research Institute, 2006502-01H; Sunnybrook Health Sciences Center, 237–2009; University of Toronto, 09-0538-BE; Maple Leaf Medical HIV Research Institute, 5005–10:221622-03-2016; Canadian Immunodeficiency Research Collaborative, 5005–14:02:0031-03-2017; CascAIDS Research Incorporated, 5005–10:58:3231-01-2017; Providence HealthCare Society, H09-01476; Interchange Medical Clinic, 5005–10:37:1824-04-2017. All patients were enrolled in the study following written informed consent. All donors were adults.

### Canadian cohort of HIV^+^ slow progressors

HLA genotyping was completed as previously described [61]. Absolute CD4^+^ and CD8^+^ T cell counts and HIV viral load were obtained at the time of blood collection [62]. Viruses were extracted from the blood of 19 donors (including 9 B27/B57^+^ individuals and 10 non-B27/B57 non-treated viremic progressors) from the Canadian Slow Progressors cohort (S1 Table and S2 Fig) [62]. We incorporated 4 additional viruses, including NRC2 and NRC10 that were isolated from B27/B57^+^ individuals, NRC1 isolated from a normal progressor and the laboratory-adapted strain NL4-3 [39, 63] (S1 Table).

### Cell culture

Jurkat, THP-1 and HEK293T (293T) cells (obtained from J. Luban, University of Massachusetts Medical School) were maintained in RPMI 1640 medium (HyClone, Thermo Scientific, USA). CRFK were maintained in DMEM medium (HyClone, Thermo Scientific, USA). All culture media were supplemented with 8% fetal bovine serum (FBS), penicillin-streptomycin (HyClone) and Plasmocin (InvivoGen).

### Chimeric vectors construction and virus production

The replication-incompetent pNL4-3_GFPΔEnvΔNef_ (thereafter called pNL4-3_GFP_) has a deletion causing a frameshift in *env* and *gfp* in place of *nef* [64]. pNL4-3_DsRed_ was constructed by replacing GFP with DsRed using NotI and XhoI in pNL4-3_GFP_. Patient blood was collected in EDTA-containing tube and HIV-1 RNA was extracted from plasma using the QIAamp Viral RNA mini kit (Qiagen). CA was amplified by RT-PCR using the SuperScript III One-Step RT-PCR System (Life Technologies) and the following cycling settings: 30 min at 55°C; 2 min at 94°C; 40 cycles (15 sec at 94°C, 30 sec at 55°C, 3 min at 68°C) with primers 5' NL4-3P24FOR and 3’ p24-1084-Rev (S4 Table). Final primer concentration was 200 nM (primer sequences are listed in S4 Table). Alternatively, whenever no CA signals could be obtained, whole *gag* sequences were amplified using primers 5’ GAGFOR692BSSHII and 3’ HIVGAG2827 REV (S4 Table) followed by nested PCR on CA using Velocity (Bioline) with the following cycling settings: 2 min at 98°C; 30 cycles (2 min at 98°C 2 min, 30 sec at 55°C, 3 min at 72°C). The 734 bp CA amplicons were then purified from agarose gels. Separately, two Gag segments upstream and downstream of CA were amplified from pNL4-3 using primer pairs 5’ GAGFOR692BSSHII and 3' NL4-3_GAGBEFOREP24 REV (487 bp upstream segment) or 5’ p24downstreamFor and 5' NL4-3GAG_AFTERGAGAPAI_REV (196 bp downstream segment) using the same cycling parameters and gel purified. Finally, the three PCR products were mixed and diluted, and a final PCR of 25 cycles was conducted using 5’ GAGFOR692BSSHII and 3’ GAGREV1959APAI. Following purification, the 1335 bp PCR products were digested with BssHII and ApaI and separated on agarose gel. Products were purified again and ligated into pNL4-3_DsRed_ or pNL4-3_GFP_ cut with ApaI and BssHII, using the T4 DNA ligase (New England Biolabs) for 10 min at room temperature followed by 16 h at 16°C. Following JM109 bacterial electroporation and culture, plasmid DNA was purified using EZ-10 Spin Column Plasmid DNA Miniprep Kit (BioBasic) and the Gag region was Sanger sequenced. Plasmid DNA was then prepared from the same bacterial clones using a Qiagen MidiPrep kit and co-transfected into 293T cells in 10 cm culture dishes at 90% confluence together with the VSV-G-expressing pMD2.G using polyethylenimine (PEI; polysciences, Niles, IL) [65]. Medium was changed 6 h post-transfection. Virus-containing supernatants were harvested 24 and 48 h later, pooled, clarified by centrifuging 10 min at 3000 rpm, 0.45 μm-filtered and stored at -80°C. The multiplicity of infection (MOI) of the HIV-1 chimeric vector particles was assessed by titration in permissive cat CRFK cells.

Capsid sequences were amplified from NL4-3, NRC10, EC5-2, EC8-2 and EC9-2 using FOR_PsPAX2_ClaI and REV_PsPAX2_ECORV were ligated into psPAX2 (Addgene #12260) cut with EcoRV and ClaI. “Empty” viral particles were constructed by co-transfection of 293T cells with psPAX2 and pMD2G using the same procedure as that described above for NL4-3-based vectors. Amounts of psPAX2-based and NL4-3 chimeric vectors were normalized by reverse transcriptase assay using the EnzChek kit (Molecular Probes).

### Knockout and knockdown constructions and transductions

The lentiviral expression vector plentiCRISPRv2 (pLCv2) was a gift from Feng Zhang (Addgene plasmid 52961) and was used to simultaneously express the gRNA, Cas9 nuclease, and *Puro*^*R*^ in THP-1 and Jurkat cell lines by lentiviral transduction [47]. gRNAs targeting exon 1 (S5 Table and S5A Fig) were designed according to Zhang’s protocol and inserted into pLCv2 leading to pLCv2-T5gRNA1 and pLCv2-T5gRNA2. Viral particles were produced by co-transfection of pLCv2, pMD.G and pΔR8.9 in 293T cells [66]. T5gRNA2 was selected to knock out *TRIM5* based on Surveyor assay results (S5B Fig). A control gRNA targeting the CAG hybrid promoter [67] was used. *TRIM5* alleles were amplified from cells transduced with pLCv2-CAG or pLCv2-T5gRNA2 treated for 10 days with puromycin (1μg/ml; Invivogen), sequenced and submitted to the *in silico* TIDE assay which quantitates percentages of indels by sequencing decomposition, in comparison with the unedited control (S5C, S5D and S5E Fig) [68]. *TRIM5* knockout was also validated by assessing N-MLV restriction for TRIM5α (S5F Fig)

Knockdowns were obtained using the pAPM-based miR30 shRNA system [33]. pAHM was generated by removal of Puro^R^ gene from pAPM by digestion with XbaI and NotI and replacement by Hygro^R^ amplified from pMIH [69] using XbaI_PAHM FOR and NOTI_PAHM REV (S5 Table). miR30-based shRNAs were designed using the publicly available Katahdin algorithm (http://katahdin.cshl.org/siRNA/RNAi.cgi?type=shRNA) and their sequences are indicated in S5 Table. An irrelevant shRNA (Luc) was used as a control. The 97 bp miR30 sequences were synthesized by Genscript (NJ, USA), amplified by PCR using the primer pairs indicated in S3 Table, digested with EcoRI and XhoI and inserted into pAHM cut with the same enzymes. shRNA sequences were verified by Sanger sequencing. pAHM was cotransfected with pΔR8.9 and pMD2.G in 293T cells. Lentiviral particles were harvested from supernatants as described above. THP-1 and Jurkat cells were spinfected in presence of polybrene (8 μg/ml) for 1.7 h at 1800 rpm. Cells were allowed to rest for 72 h and treated with 250 μg/ml hygromycin (Sigma) for 10 days. Knockdown efficiency was verified using mRNA transcript levels quantification by RT-qPCR.

### RT-qCPR

TRIzol (Life Technologies) and chloroform (Sigma-Aldrich) were used to extract total RNA from cultured cells. Glycogen (Life Technologies) was added during the extraction to enhance RNA yields and cDNA was synthesized using the SuperScript IV (Life technologies) according to the manufacturer’s protocol. Amplification was performed using 400 nM of forward and reverse primers (S6 Table), and 5 μl template (150–500 ng) in 10 μl final volume according to the SensiFast SYBR Lo-ROX kit protocol (Bioline). After 3 min incubation at 95°C, 40 cycles of amplification were performed as follows: 5 sec at 95°C, 10 sec at 60°C, 15 sec at 72°C. Each PCR was performed in duplicate and the threshold cycle (C_t_) was determined using the MxPro software (Agilent). Relative expression was calculated using the ΔCt method with GAPDH for normalization (2^– (*C*t(target)-*C*t(*GAPDH*))^).

### Viral challenges and flow cytometric analyses

To measure chimeric HIV-1 vectors sensitivity to TRIM5α, *TRIM5* knockout and control cells were seeded into 96-well plates at 4 × 10^4^ cells/well and recombinant human IFN-β was added at a final concentration of 10 ng/ml (PeproTech, Rocky Hill, NJ). Cells were infected the following day with serial 2-fold dilutions of DsRed- or GFP-expressing chimeric vectors at MOIs ranging from 0.1 to 2.5. The percentage of DsRed- or GFP-positive cells was determined after 48–72 h of infection. For this, cells were fixed in 4% formaldehyde (Fisher Scientific, MA, USA) and 1×10^4^ to 5×10^4^ cells were analyzed on a FC500 MPL cytometer (Beckman Coulter, Inc., CA) using the FCS express 6 software (De novo software, CA). The -fold restriction was calculated as the mean ratio of viral titers between TRIM5 knockout and control cells (titer calculations only took into account the vector amounts leading to percentages of infected cells between 0.5 and 10). Infections were repeated multiple times and mean -fold restrictions were calculated.

To quantify the antiviral state, TRIM5 KO and control (CAG) cells were seeded into 96-well plates at 4×10^4^ cells/well and infected the next day with the DsRed-chimeric vectors (virus 1) at a CRFK MOI of 0.25–0.5 in Jurkat and 1–2.5 in THP-1 cells. Where indicated, cells were pre-treated for 45–60 minutes prior to the first infection with inhibitors of NF-κB (BAY11-7085; ENZO) or cJun (SP600125; ENZO), or TBK1/IKKε (BX795, Tocris), or with the blocking antibody anti-IFNα/βR2 20 μg/ml (clone MMHAR-2; PBL Assay Science). 60-minutes pre-treatments were also done with Raltegravir (20 μM; Merck). Pre-treated cells were thoroughly washed prior to the first infection in order to remove the pharmacological inhibitors. Where specified, viruses were pre-treated with 300 μM Aldrithiol-2 (prepared in MetOH; Sigma) for 2 h at 4°C or with MetOH only, with shaking [50]. AT-2 and MetOH-treated virions were then diluted 10 times, ultracentrifuged for 90 min at 20,000 g and resuspended in PBS. The absence of infectivity following AT-2 treatment was verified by FACS. 48 h post-infection with virus 1, virus 2 (NRC1_GFP_ or NRC10_GFP_) was added to the cells at an MOI similar to virus 1. Two days later, cells were fixed in 3% formaldehyde and analyzed by FACS. Infection experiments were performed in triplicates.

### Immunofluorescence microscopy

TRIM5-KO and control THP-1 cells were treated with 100 nM of Phorbol 12-myristate 13-acetate (PMA, Sigma-Aldrich) for 24 h while seeded on glass coverslips, then washed and placed in normal medium. After 72 h, differentiated THP-1 were infected with NRC1_GFP_ or NRC10_GFP_ for 72 h. Cells were then fixed and permeabilized as published previously [65], and incubated with anti-P-p65 or anti-P-cJun (1:150 in 10% bovine serum; Cell Signaling) at RT for 4 h. Following 4 PBS washes, cells were stained with Alexa Fluor 594-conjugated goat anti-rabbit (Molecular Probes, Eugene, OR) diluted 1:100 in 10% bovine serum for 1 h at RT. Slides were mounted as previously described [65] and pictures were acquired on the Axio Observer microscope (Carl Zeiss, Inc., Toronto, ON, Canada).

### ELISA

IFN-β was quantified in culture supernatants 72 h post-infection using the Verikine High Sensitivity Human IFN-β ELISA kit according to the manufacturer’s instructions (PBL IFN Source).

### Statistical analyses

The GraphPad Prism software was used for statistical tests and for generating graphs. Non-parametric tests were used when data did not fit Gaussian distribution.

## Supporting information

S1 Table Patient data. (PDF)

S2 Table CA N-terminal amino acid sequences from clinical isolates. (PDF)

S3 Table CA polymorphisms and associated functions in published reports. (PDF)

S4 Table Oligodeoxynucleotide (ODN) primers used for Gag amplification and cloning. (PDF)

S5 Table ODN primers used to construct vectors for the expression of gRNAs and shRNAs. (PDF)

S6 Table ODN primers used for Real-Time qPCR. (PDF)

S1 References References for supplementary tables. (PDF)

S1 Fig Patient characteristics. **(a)** CD4+ T cell counts, **(b)** viremia, at the time-points used in this study and according to their HLA type. B27 or B57 are grouped together. **(c)** Evolution of CD4, CD8 and virus counts for EC9. The black arrow indicates the time-point used in this study. The red line shows initiation and continuation of antiretroviral therapy.
(PDF)

S2 Fig HLA status and presence of mutations known to modulate restriction by Mx2 and TRIM5α. **(a)** Bar graph of the contingency table of mutations at the G116 position (Mx2) in individuals bearing B27/B57 or other alleles (Chi-square test; *p* = 0.0169). **(b)** Bar graphs showing the presence or the absence of mutations previously shown to be associated with TRIM5α sensitivity according to the HLA status (Fisher’s exact test; *p* = 0.0412).
(PDF)

S3 Fig TRIM5α and Mx2 knockdown validation. **(a)** mRNA levels were determined by RT-qPCR and normalized on GAPDH mRNA levels. Shown are mean mRNA levels calculated by RT-qPCR performed in duplicates on total RNA extracted from IFN-β-treated Jurkat cells, and normalized to the shLuc control. **(b)** Same analysis in THP-1 cells.
(PDF)

S4 Fig Sensitivity of NRC10_GFP_ and NL43_GFP_ to restriction by Mx2 and TRIM5α. Jurkat cells knocked down for Mx2 or TRIM5α or both were infected with increasing amounts of the two HIV-1vectors. Infectivity was measured by FACS as the % of GFP^+^ cells 48 h post-infection.
(PDF)

S5 Fig CRISPR/Cas9-mediated editing of TRIM5α in human cell lines. **(a)** Cas9 was targeted to exon 1 of the TRIM5 gene (green) by two selected gRNAs, whose binding sites are schematized with scissors. Arrowheads indicate the positions of the binding sites for the ODNs used in the PCR-Surveyor assay. **(b)** Surveyor assay. Briefly, PCR products amplified from 293T cells transfected with pLCv2-hT5g1, pLCv2-hT5g2, or pLCv2-CAG (control) were subjected to denaturation, reannealing, and digestion with the Surveyor enzyme. Arrows indicate cleavage products of the expected size. **(c)** Sanger sequencing analysis. THP-1 cells were transduced with lentiviral vectors produced using pLCv2-hT5g2 or the control vector, pLCv2-CAG. Following puromycin selection, the targeted *TRIM5* locus was PCR amplified and the PCR product was Sanger sequenced. The figure shows an alignment of the obtained sequence plots. **(d, e)** Decomposition of sequencing plots by TIDE assay. The graphs on the left show the percentages of aberrant peaks upstream and downstream of the cut site in the sequencing reactions shown in panel c in THP-1 (d) and in Jurkat (e) cells. The graphs on the right display the frequency of aberrant sequence signals in *TRIM5* sequences in corresponding T5KO (test sequences in green) and control (CAG in black) cells. The percentage of indel-containing alleles was computed by the TIDE assay. **(f)** T5KO and control THP-1 were infected with N-MLV_GFP_ and B-MLV_GFP_. Infectivity was assessed by flow cytometry 72 h post-infection. **(g)** Knockdown validation for Ubc13 and TAK1 in TRIM5α knockout and control cells. mRNA levels were determined by RT-qPCR and normalized on GAPDH mRNA levels. Shown are mean mRNA levels calculated by RT-qPCR performed in duplicates on total RNA extracted from TRIM5-KO and control CAG THP-1 and Jurkat cells as indicated.
(PDF)

S6 Fig Effect of inhibitors on HIV-1 vector infectivity. THP-1 cells were pre-treated or not with **(a)** BX795 (iTBK1), **(b)** BAY11-7085 (iNF-κB) or **(c)** SP600125 (iAP-1) for 1 h, infected with DsRed-expressing chimeric vectors (“virus 1”), and 48 h later infected with NRC1_GFP_ (“virus 2”). Infectivity of DsRed-virus 1 was assessed by flow cytometry 48 h later. Data are from the same infections as those shown in Fig 5C, Fig 6D and Fig 6E, respectively.
(PDF)

S7 Fig Productively infected cells in microscopy experiments. **(a)** Microscopy images corresponding to Fig 6A with the GFP field included. **(b)** Frequency of infected (GFP^+^) cells quantified by analyzing ≥100 cells from ≥ 10 pictures and plotted according to TRIM5α expression and viral infection. The Kruskal-Wallis test and the Dunn's Multiple Comparison Test were used to assess statistical significance. Shown are means with SEM. noV = No virus, Vir = virus, RAL = Raltegravir, EV = empty vector. **(c)** T5KO and control THP-1 cells were treated for 60 min with Raltegravir (RAL) or left untreated then infected with the GFP-expressing EC5-2 vector at a CRFK MOI = 2. Infectivity (% GFP^+^ cells) was measured by FACS at 48 h post-infection.
(PDF)

S8 Fig Hypothetical model. Following entry, viruses from a B27/B57^+^ subject escape Mx2 restriction but are recognized by TRIM5α. TRIM5α disrupts the proper uncoating process and may trigger pro-inflammatory signals through Ubc13- and TAK1-dependent signaling. In THP-1 cells, this leads to activation of NF-κB and AP-1 and production of type I IFN production that signals through IFNAR1/2 to induce an antiviral state that blocks infection from TRIM5α-sensitive as well as TRIM5α-resistant HIV-1 viruses. In Jurkat cells, the antiviral state is induced in an IFN-I-independent manner.
(PDF)
