# Supplementary material for: HIV-1 capsids from B27/B57+ elite controllers escape Mx2 but are targeted by TRIM5α, leading to the induction of an antiviral state
Source: PLoS Pathog. 2018 Nov 12;14(11):e1007398. doi: 10.1371/journal.ppat.1007398 (PMC6258467; doi:10.1371/journal.ppat.1007398)
Supplement: S1 Table — (PDF) [file ppat.1007398.s001.pdf]

**Table S1.** Patient data.

| Patients | HLA | CD4 count | CD8 count | Viremia (U/ml) |
|---|---|---|---|---|
| **EC1** | B07/B57 | 420 | 530 | 40 |
| **EC2** | B13/B57 | 490 | 870 | 40 |
| **EC3** | B14/B27 | 670 | 630 | 50 |
| **EC4** | B15/ B27 | 620 | 500 | 40 |
| **EC5** | B15/B27 | 580 | 340 | 50 |
| **EC6** | B18/B27 | 990 | 1170 | 50 |
| **EC8** | B35/B57 | 1130 | 1117 | 40 |
| **EC9** | B57/B57 | 238 | nd | 9239 |
| **EC7** | B57/B58 | 576 | 990 | 40 |
| **NRC2** | B57/B40 | nd | nd | nd |
| **NRC10** | B27/B55 | nd | nd | nd |
| **NP1** | B14/B18 | 260 | 550 | 2508 |
| **NP2** | B35/B39 | 440 | 1390 | 137715 |
| **NP3** | B07/B08 | 460 | 1030 | 123474 |
| **NP4** | B07/B53 | 430 | 1130 | 296041 |
| **NP5** | B08/B14 | 450 | 590 | 2819 |
| **NP6** | B38/B39 | 390 | 590 | 1597 |
| **NP7** | B14/B14 | 560 | 770 | 2767 |
| **NP8** | B14/B35 | 870 | 1400 | 19311 |
| **NP9** | B07/B44 | 360 | 760 | 88455 |
| **NP10** | B07/B18 | 610 | 690 | 41285 |
| **NRC1** | B14/B44 | nd | nd | nd |

*nd, not determined. Accession numbers: NRC1, JN408075; NRC2, JN408076.1; NRC10, JN408077.1 (1). NL4-3 Gag is derived from NY5, accession number AF324493 (2). Patient HLA, CD4 and CD8 counts and viremia were determined at the time of isolate extraction.
