# Supplementary material for: HIV-1 capsids from B27/B57+ elite controllers escape Mx2 but are targeted by TRIM5α, leading to the induction of an antiviral state
Source: PLoS Pathog. 2018 Nov 12;14(11):e1007398. doi: 10.1371/journal.ppat.1007398 (PMC6258467; doi:10.1371/journal.ppat.1007398)
Supplement: S2 Table — (PDF) [file ppat.1007398.s002.pdf]

Table S2. CA N-terminal amino acid sequences from clinical isolates.

	133 146 IW9 159 162 KF11 173 215 CYPA 228 238 TW10 262 KK10	Restriction[1]
**NL43**	PIVQNL...AISPRTLNAWVKVVEEKAFSPEVIPMFS...LHPVHAGPIAPGQMR...GTTSTLQEQIGWM...YKRWIILGLNK	1.8 ; 1.9
**NRC1**	-----A...-L-------------------------S...---AQ---V----L-...----N-----A--...------M----	1.7 ; 1.3
**NRC2**	------...PL---------------G-N-------S...--------V------...----N-----A--...-----------	4.2 ; 3.5
**NRC10**	------...-------------I-------------T...---A---------I-...----------A--...--K--VM----	12.5 ; 5.4
**EC1**	----D-...PL-------------------------S...---------------...----N--------...-----------	ND
**EC3**	------...-------------I-------------S...---L---------I-...----------R--...-----------	9.1 ; 3.6
**EC5-1**	------...PL-----------I-------------T...--------A------...----------A--...-----------	5.6 ; 3.8
**EC5-2**	------...PL-----------I-------------S...--------A------...----------A--...-----------	5.1 ; 3.0
**EC6**	---H--...VM-----------I-------------T...---------------...----------A--...------M----	1.9 ; 2.5
**EC7**	------...--A----------I-------------T...---PQ---V----L-...----N-----Q--...-----VM----	1.3 ; 1.7
**EC8-1**	-----T...PL-----------I-------------S...--------A------...----N--------...-----------	NI
**EC8-2**	------...PL---------------S---------S...--------A------...----------A--...-----------	9.5 ; 4.9
**EC8-3**	-----T...PL-----------I-------------S...--------A------...----------A--...-----------	9.0 ; 4.6
**EC9-1**	------...--A----------I-------------T...---PQ---V----I-...----NP----Q--...-----VM--S-	24.5 ; 6.2
**EC9-2**	------...--A----------I-------------T...---AQ---V----I-...----N-----Q--...--S--VM----	4.5 ; ND
**EC9-3**	------...--A----------I-------------S...----Q---V----I-...-------------...--G--------	NI
**EC9-4**	------...P-A----------I-------------T...---PQ--------I-...----N-----Q--...-----VM----	3.0 ; 3.7
**EC9-5**	------...--A----------I-K-----------T...---PQ---V----I-...----N-----Q--...-----VM----	4.7 ; ND
**NP1**	------...--A----------I-------------T...---PQ---V----L-...----N-----Q--...-----VM----	1.6 ; 0.9
**NP2**	-----M...PL-----------I-------------T...---------------...-------------...-----------	1.8 ; 0.7
**NP3**	------...---------------------------S...---------------...-------------...-----------	4.2 ; 2.1
**NP4**	------...---------------------------S...---------------...-------------...-----------	2.8 ; 1.9
**NP5**	------...-------------I-------------S...V--------------...-------------...-----------	NI
**NP6**	------...-L-------------------------T...----Q---V------...-------------...------M----	1.3 ; ND
**NP8**	------...P--------------------------A...---------------...-------D-----...--K---M----	1.3 ; 1.8
**NP10**	------...P--------------------------A...---------------...-------------...--K---M----	1.2 ; 1.2

[1]TRIM5α restriction activity was measured in THP-1 and Jurkat cells as described in Methods. NI, not infectious; ND, not determined. Underlined in the NL43 sequence are the immunodominant epitopes (IW9, KF11, CYPA, TW10 and KK10) commonly presented by B27 or B57 alleles. Polymorphisms previously described as $CD8^+$ T cell-escape mutations (1, 3-5) are shown in red. Polymorphisms previously proposed to be CTL escape compensatory mutations are in green. Polymorphisms of unknown function/origin or that have not been previously described are shown in blue. Mutations previously identified as determinants for the increased sensitivity of NRC2 and NRC10 to TRIM5α are in bold.
