# Supplementary material for: HIV-1 capsids from B27/B57+ elite controllers escape Mx2 but are targeted by TRIM5α, leading to the induction of an antiviral state
Source: PLoS Pathog. 2018 Nov 12;14(11):e1007398. doi: 10.1371/journal.ppat.1007398 (PMC6258467; doi:10.1371/journal.ppat.1007398)
Supplement: S3 Table — (PDF) [file ppat.1007398.s003.pdf]

Table S3. CA polymorphisms and associated functions in published reports.

Mutation	Role	Ref	Isolate ID
**M10I**	- Resistant to Mx2<br>- Resistant to rhTRIM5α	(6, 7)	none
**M10L**	- Resistant to rhTRIM5α	(7)	EC5, EC6, EC8
**N5D (N137D)**	- Unknown	n/a	EC1
**L6A (L138A)**	- Sensitive to huTRIM5α	(8)	NRC1
**L6T**	- Sensitive to huTRIM5α	(8)	EC7, EC8
**L6M**	- Sensitive to huTRIM5α	(8)	NP2
**A14P (A146P)**	- ISW9/CTL escape mutation	(9)	NRC2, EC1, EC5, EC8, EC9, NP2, NP8
**A14V**	- ISW9/CTL escape mutation	(9)	EC6
**I15L (I147L)**	- ISW9/CTL escape mutation	(9, 10)	NRC1, NRC2, EC1, EC5, EC8, NP2, NP6
**I15M**	- ISW9/CTL escape<br>- HL9/CTL escape (A30/B13/C6)	(10)	EC6
**S16A(S148A)**	- ISW9/CTL escape mutation<br>- HL9/CTL escape (A30/B13/C6)	(10)	EC7, EC9, NP1
**V27I (V159I)**	- HLA-B57/58:01-associated mutation	(11)	NRC10, EC3, EC5, EC6, EC7, EC8, EC9, NP1, NP2, NP5
**A31G (A163G)**	- Epitope KF11/CTL escape<br>- 'Footprint B57'	(12)	NRC2
**A31S**	- Epitope KF11/CTL escape<br>- Loss of fitness	(9, 13)	EC8
**S33N (S165N)**	- Epitope KF11/CTL escape<br>- 'Footprint B57'	(12)	NRC2
**P38A (P170A)**	- Unstable core<br>- Resistant to Mx2	(14)	none
**S41T (S173T)**	- Epitope KF11<br>- Compensation to CTL escape R132K (R264K)<br>- Sensitive to huTRIM5α	(1, 5)	NRC10, EC5, EC6, EC7, EC9, NP1, NP2, NP6
**S41A**	- Compensation to CTL escape R132K (R264K)<br>- Resistance to CPSF6-mediated restriction	(5, 15)	EC8
**E45A (E177A)**	- Increased core stability<br>- Not infectious in nondividing cells	(16, 17)	none
**T54A (T186A)**	- Decreased core stability<br>- Altered sensitivity to CypI	(18)	none
**N57S (N189S)**	- Resistant to Mx2<br>- Resistant to rhTRIM5α	(6, 7, 19)	none
**Q63A(Q195A)**	- Decreased core stability<br>- Resistant to Mx2<br>- Not infectious in nondividing cells	(14, 17)	none
**Q67A(Q199A)**	- Decreased core stability<br>- Resistant to Mx2<br>- Can not infect nondividing cells	(14)	none
**N74D (N206D)**	- Resistant to Mx2<br>- Increased IFN production<br>- CPSF6 independent	(15, 20)	none
**N74K**	unknown	n/a	NP4
**E79D**	- Resistant to Mx2	(21)	none
**L83V(L215V)**	unknown	n/a	NP5
**L83T**	- Resistant to Mx2		none
**V86A (V218A)**	- CypA binding loop<br>- Compensatory mutation<br>- Sensitive to huTRIM5α<br>- Resistant to Mx2	(1, 22, 23)	NRC1, NRC10, EC9
**V86L**	unknown	n/a	EC3
**V86P**	- Resistant to CypI	(24)	EC9, NP1
**H87P(H219P)**	- Resistant to IFN<br>- Resistant to Mx2	(14)	none
**H87R**	- Resistant to Mx2	(6)	none
**H87Q**	- CypA binding loop<br>- Resistant to CypI<br>- CTL escape/B57 footprint<br>- Resistant or sensitive to huTRIM5α<br>- Resistant or sensitive to Mx2	(8, 21, 23, 25-27)	NRC1, EC7, EC9, NP6
**G89A/V (G221A)**	- Resistant to Mx2<br>- Increased IFN production	(14, 19)	none
**P90A/T (P222A)**	- Resistant to Mx2<br>- Increased IFN production (cGAS)	(6, 20)	none
**I91V(I223V)**	- CypA binding loop<br>- Resistant to CypI<br>- CTL escape/B57 footprint<br>- Sensitive to huTRIM5α	(8, 28)	NRC1, NRC2, EC5, EC8

**A92P**	- Resistant to Mx2	(23)	none
**Q95L (Q227V)**	- Resistant to Mx2	(6)	none
**M96L(M228L)**	- CypA binding loop<br>- Resistant to CypI<br>- CTL escape/B57 footprint	(28)	NRC1, EC7, EC9
**M96I**	- CypA binding loop<br>- Resistant to CypI<br>- CTL escape<br>- Sensitive to huTRIM5α	(1, 24)	NRC10, EC9, EC3
**A105T (A237T)**	- TW10 epitope<br>- Resistant to CypI<br>- Sensitive to IFN	(18, 20)	none
**T110N (T242N)**	- TW10 epitope<br>- Low infectivity/unstable core<br>- Sensitive to huTRIM5α<br>- CTL escape/B57 footprint	(1, 4)	NRC1, NRC2, EC1, EC7, EC8, EC9, NP6
**L111P**	- TW10 epitope	n/a	EC9
**E113D**	- TW10 epitope	n/a	NP8
**G116A**	- TW10 epitope<br>- CTL escape/B57 footprint<br>- Sensitive to huTRIM5α<br>- Resistant to Mx2	(1, 14)	NRC1, NRC2, NRC10, EC5, EC6, EC8
**G116R**	- TW10 epitope		EC3
**G116Q**	- TW10 epitope		EC7, EC9, NP1
**N121S**	- Resistant to CypI	(29)	none
**R132K (R264K)**	- KK10 epitope/CTL escape<br>- Resistant to CypI<br>- Decreased infectivity<br>- Sensitive to huTRIM5α	(1)	NRC10, NP8
**R132S**	- Unstable core	(5)	EC9
**R132G**	unknown	n/a	EC9
**I135V (I267V)**	- KK10 epitope	n/a	NRC10, EC7, EC9, NP1
**L136M (L268M)**	- KK10 epitope/CTL escape<br>- Decreased infectivity<br>- Sensitive to huTRIM5α	(1)	NRC1, NRC10, EC6, EC7, EC9, NP1, NP6, NP8
**M185I**	- Resistant to Mx2	(6)	none
**E187V**	- Resistant to AgMx2	(6)	none
**E187G**	unknown		EC9, NP5
**K203A**	- Unstable core	(30)	none
**P207A/R/S/T**	- Resistant to Mx2<br>- Sensitive to CSA	(6, 14)	none
**G208R**	- Resistant to Mx2<br>- Sensitive to CSA	(6)	none
**G208A**	unknown	n/a	EC7, EC9
**T210K**	- Resistant to Mx2<br>- Sensitive to CSA	(6)	none
**T210S**	unknown	n/a	EC8

‘Sensitive to huTRIM5α’ means that a more potent restriction was observed than for the wild-type virus. Conversely, ‘resistant to Mx2’ or rhT5 or CypI (Cyclophilin A inhibitors) means that the corresponding virus was less sensitive to inhibition than its wild-type counterpart.
