# Supplementary material for: HIV-1 capsids from B27/B57+ elite controllers escape Mx2 but are targeted by TRIM5α, leading to the induction of an antiviral state
Source: PLoS Pathog. 2018 Nov 12;14(11):e1007398. doi: 10.1371/journal.ppat.1007398 (PMC6258467; doi:10.1371/journal.ppat.1007398)
Supplement: S4 Table — (PDF) [file ppat.1007398.s004.pdf]

Table S4. Oligodeoxynucleotide (ODN) primers used for Gag amplification and cloning.

Name	Sequence
**5’ GAGFOR692BSSHII**	CAGGACTCGGCTTGCTGAAGCGCGCA
**3’ GAGREV1959APAI**	AGCCCTTTTTCCTAGGGGCCCTGC
**3’ HIVGAG2827 REV**	TAACCCTGCGGGATGTGGTATTCC
**3' NL4-3_GAGBEFOREP24 REV**	TGGCTGACCTGGCTGTTGTT
**5' NL4-3P24FOR**	CAACAGCCAGGTCAGCCAAAATTA
**3’ p24-1084-Rev**	GGCTCATTGCTTCAGCCAAAA
**5’ p24downstreamFor**	GGACCCGGCCATAAAGCAAG
**5' NL43_AFTERGAGAPAI_REV**	CTTCCTTTCCACATTTCCAACAGC
**5’DsRed NotI**	GATGCGGCCGCAATGGACAACACCGAG
**3’DsRed XhoI**	GTACTCGAGCTACTGGGAGCCGGAGTG
**5’FOR_PsPAX2_ClaI**	GAGAATTAGATCGATGGGAAAAAATTCGGTTAAGGC
**3’REV_PsPAX2_ECORV**	CATTGTACTGATATCTAATCCCTGGTGTCTCATTGTTTATAC
