# Supplementary material for: HIV-1 capsids from B27/B57+ elite controllers escape Mx2 but are targeted by TRIM5α, leading to the induction of an antiviral state
Source: PLoS Pathog. 2018 Nov 12;14(11):e1007398. doi: 10.1371/journal.ppat.1007398 (PMC6258467; doi:10.1371/journal.ppat.1007398)
Supplement: S5 Table — (PDF) [file ppat.1007398.s005.pdf]

Table S5. ODN primers used to construct vectors for the expression of gRNAs and shRNAs.

Name	Target	Sequence	Reference
**gRNA#2 hT5 bottom**	TRIM5α gRNA	AAACGTGTGCCGGATCAGTTACCAC	n/a
**gRNA#2 hT5 top**	TRIM5α gRNA	CACCGTGGTAACTGATCCGGCACAC	n/a
**Control gRNA top CAG**	Ctrl gRNA	CACCGGTTCCGCGTTACATAACTTA	(31)
**Control gRNA bottom CAG**	Ctrl gRNA	AAACTAAGTTATGTAACGCGGAACC	(31)
**NOTI_PAHM REV**	Hygromycin	GCTTGCGGCCGCCTATTCCTTTGCCCTC	n/a
**XbaI_PAHM FOR**	Hygromycin	GGGGTCTAGAGCCACCATGAAAAAGCCTGAACTC	n/a
**mir30XhoIFor**	miR30	AAGGCTCGAGAAGGTATATTGCTGTTGACAGTGA G	(32)
**mir30EcoRIRev**	miR30	AGCCCCTTGAATTCCGAGGCAGTAGGCA	(32)
**shMx2**	miR30	TCAAGATGTTCTTTCTAATTGA	(33)
**shLuc**	miR30	TACAAACGCTCTCATCGACAAG	(32)
**shTAK1**	miR30	AGCGCCCTTCAATGGAGGAAAT	(32)
**shUbc13**	miR30	AGCTAACCAGGTCTTTAGAATA	(32)
**shTRIM5**	miR30	TGCCAAGCATGCCTCACTGCAA	(32)

gRNAs were designed according to Zhang’s protocol (see Methods). n/a, not applicable as they were designed in-house.
