# Supplementary material for: HIV-1 capsids from B27/B57+ elite controllers escape Mx2 but are targeted by TRIM5α, leading to the induction of an antiviral state
Source: PLoS Pathog. 2018 Nov 12;14(11):e1007398. doi: 10.1371/journal.ppat.1007398 (PMC6258467; doi:10.1371/journal.ppat.1007398)
Supplement: S6 Table — (PDF) [file ppat.1007398.s006.pdf]

Table S6. ODN primers used for Real-Time qPCR.

Name	Sequence	Ref
**5'Hu-rhTRIM5a-QPCR-UJ For**	AGACATTCTGAAAAGCCTTACGAA	(34)
**3'Hu-rhTRIM5a-QPCR-UJ-Rev**	ATCAGGAGCTCGAAACACTCTC	(34)
**5'TAK1_QPCR_FOR**	CCCAGGTCCTCAACTTTGAAG	n/a
**3'TAK1_QPCR_REV**	ATATTAGGATGGTTCACACGGG	n/a
**5'UBC13_QPCR_FOR**	AAGTGCTCCCAATCCAGATG	n/a
**3'UBC13_QPCR_REV**	TTGGCAGAACAGGAGAAGTG	n/a
**5'MX2 FWD**	AGTCTTCGGTTTCCTCCTTTA	n/a
**3'MX2 REV**	CTGCAAGGAGTCACCATTCT	n/a
**5'GAPDH-QPCR FOR**	CCACTCCTCCACCTTTGAC	n/a
**3'GAPDH-QPCR REV**	ACCCTGTTGCTGTAGCCA	n/a

n/a, not applicable as they were designed in-house.
