# Supplementary material for: HIV-1 capsids from B27/B57+ elite controllers escape Mx2 but are targeted by TRIM5α, leading to the induction of an antiviral state
Source: PLoS Pathog. 2018 Nov 12;14(11):e1007398. doi: 10.1371/journal.ppat.1007398 (PMC6258467; doi:10.1371/journal.ppat.1007398)
Supplement: S1 References — (PDF) [file ppat.1007398.s007.pdf]

S1 References

1. Battivelli E, Migraine J, Lecossier D, Yeni P, Clavel F, Hance AJ. Gag cytotoxic T lymphocyte escape mutations can increase sensitivity of HIV-1 to human TRIM5alpha, linking intrinsic and acquired immunity. Journal of virology. 2011;85(22):11846-54. Epub 2011/09/16.
2. Adachi A, Gendelman HE, Koenig S, Folks T, Willey R, Rabson A, et al. Production of acquired immunodeficiency syndrome-associated retrovirus in human and nonhuman cells transfected with an infectious molecular clone. Journal of virology. 1986;59(2):284-91.
3. Battivelli E, Migraine J, Lecossier D, Matsuoka S, Perez-Bercoff D, Saragosti S, et al. Modulation of TRIM5alpha activity in human cells by alternatively spliced TRIM5 isoforms. Journal of virology. 2011;85(15):7828-35. Epub 2011/06/03.
4. Brockman MA, Schneidewind A, Lahaie M, Schmidt A, Miura T, Desouza I, et al. Escape and compensation from early HLA-B57-mediated cytotoxic T-lymphocyte pressure on human immunodeficiency virus type 1 Gag alter capsid interactions with cyclophilin A. Journal of virology. 2007;81(22):12608-18.
5. Schneidewind A, Brockman MA, Yang R, Adam RI, Li B, Le Gall S, et al. Escape from the dominant HLA-B27-restricted cytotoxic T-lymphocyte response in Gag is associated with a dramatic reduction in human immunodeficiency virus type 1 replication. Journal of virology. 2007;81(22):12382-93.
6. Busnadiego I, Kane M, Rihn SJ, Preugschas HF, Hughes J, Blanco-Melo D, et al. Host and viral determinants of Mx2 antiretroviral activity. Journal of virology. 2014;88(14):7738-52. Epub 2014/04/25.
7. Soll SJ, Wilson SJ, Kutluay SB, Hatziioannou T, Bieniasz PD. Assisted evolution enables HIV-1 to overcome a high TRIM5alpha-imposed genetic barrier to rhesus macaque tropism. PLoS pathogens. 2013;9(9):e1003667.
8. Rahm N, Gfeller D, Snoeck J, Martinez R, McLaren PJ, Ortiz M, et al. Susceptibility and adaptation to human TRIM5alpha alleles at positive selected sites in HIV-1 capsid. Virology. 2013;441(2):162-70. Epub 2013/04/23.
9. Crawford H, Lumm W, Leslie A, Schaefer M, Boeras D, Prado JG, et al. Evolution of HLA-B*5703 HIV-1 escape mutations in HLA-B*5703-positive individuals and their transmission recipients. J Exp Med. 2009;206(4):909-21.
10. Zhang H, Han X, Zhao B, An M, Wang Z, Jiang F, et al. Multilayered HIV-1 gag-specific T-cell responses contribute to slow progression in HLA-A*30-B*13-C*06-positive patients. Aids. 2015;29(9):993-1002.
11. Gijsbers EF, Feenstra KA, van Nuenen AC, Navis M, Heringa J, Schuitemaker H, et al. HIV-1 replication fitness of HLA-B*57/58:01 CTL escape variants is restored by the accumulation of compensatory mutations in gag. PLoS One. 2013;8(12):e81235.
12. Matthews PC, Leslie AJ, Katzourakis A, Crawford H, Payne R, Prendergast A, et al. HLA footprints on human immunodeficiency virus type 1 are associated with interclade polymorphisms and intraclade phylogenetic clustering. Journal of virology. 2009;83(9):4605-15.
13. Pohlmeyer CW, Buckheit RW, 3rd, Siliciano RF, Blankson JN. CD8+ T cells from HLA-B*57 elite suppressors effectively suppress replication of HIV-1 escape mutants. Retrovirology. 2013;10:152.
14. Wei W, Guo H, Ma M, Markham R, Yu XF. Accumulation of MxB/Mx2-resistant HIV-1 Capsid Variants During Expansion of the HIV-1 Epidemic in Human Populations. EBioMedicine. 2016;8:230-6.

15. Henning MS, Dubose BN, Burse MJ, Aiken C, Yamashita M. In vivo functions of CPSF6 for HIV-1 as revealed by HIV-1 capsid evolution in HLA-B27-positive subjects. PLoS Pathog. 2014;10(1):e1003868.
16. von Schwedler UK, Stemmler TL, Klishko VY, Li S, Albertine KH, Davis DR, et al. Proteolytic refolding of the HIV-1 capsid protein amino-terminus facilitates viral core assembly. The EMBO journal. 1998;17(6):1555-68.
17. Yamashita M, Emerman M. Cellular restriction targeting viral capsids perturbs human immunodeficiency virus type 1 infection of nondividing cells. Journal of virology. 2009;83(19):9835-43.
18. Yang R, Aiken C. A mutation in alpha helix 3 of CA renders human immunodeficiency virus type 1 cyclosporin A resistant and dependent: rescue by a second-site substitution in a distal region of CA. Journal of virology. 2007;81(8):3749-56.
19. Kane M, Yadav SS, Bitzegeio J, Kutluay SB, Zang T, Wilson SJ, et al. MX2 is an interferon-induced inhibitor of HIV-1 infection. Nature. 2013;502(7472):563-6. Epub 2013/10/15.
20. Bulli L, Apolonia L, Kutzner J, Pollpeter D, Goujon C, Herold N, et al. Complex Interplay between HIV-1 Capsid and MX2-Independent Alpha Interferon-Induced Antiviral Factors. Journal of virology. 2016;90(16):7469-80. Epub 2016/06/10.
21. Nakayama EE, Saito A, Sultana T, Jin Z, Nohata K, Shibata M, et al. Naturally occurring mutations in HIV-1 CRF01_AE capsid affect viral sensitivity to restriction factors. AIDS research and human retroviruses. 2018.
22. Aberham C, Weber S, Phares W. Spontaneous mutations in the human immunodeficiency virus type 1 gag gene that affect viral replication in the presence of cyclosporins. Journal of virology. 1996;70(6):3536-44.
23. Liu Z, Pan Q, Liang Z, Qiao W, Cen S, Liang C. The highly polymorphic cyclophilin A-binding loop in HIV-1 capsid modulates viral resistance to MxB. Retrovirology. 2015;12(1):1.
24. Gallay PA, Bobardt MD, Chatterji U, Trepanier DJ, Ure D, Ordonez C, et al. The Novel Cyclophilin Inhibitor CPI-431-32 Concurrently Blocks HCV and HIV-1 Infections via a Similar Mechanism of Action. PLoS One. 2015;10(8):e0134707.
25. Gallay PA, Ptak RG, Bobardt MD, Dumont JM, Vuagniaux G, Rosenwirth B. Correlation of naturally occurring HIV-1 resistance to DEB025 with capsid amino acid polymorphisms. Viruses. 2013;5(3):981-97.
26. Gatanaga H, Das D, Suzuki Y, Yeh DD, Hussain KA, Ghosh AK, et al. Altered HIV-1 Gag protein interactions with cyclophilin A (CypA) on the acquisition of H219Q and H219P substitutions in the CypA binding loop. The Journal of biological chemistry. 2006;281(2):1241-50.
27. Ikeda Y, Ylinen LM, Kahar-Bador M, Towers GJ. Influence of gag on human immunodeficiency virus type 1 species-specific tropism. Journal of virology. 2004;78(21):11816-22.
28. Chatterji U, Bobardt MD, Stanfield R, Ptak RG, Pallansch LA, Ward PA, et al. Naturally occurring capsid substitutions render HIV-1 cyclophilin A independent in human cells and TRIM-cyclophilin-resistant in Owl monkey cells. The Journal of biological chemistry. 2005;280(48):40293-300.
29. Matsuoka S, Dam E, Lecossier D, Clavel F, Hance AJ. Modulation of HIV-1 infectivity and cyclophilin A-dependence by Gag sequence and target cell type. Retrovirology. 2009;6:21. Epub 2009/03/04.

30. Forshey BM, von Schwedler U, Sundquist WI, Aiken C. Formation of a human immunodeficiency virus type 1 core of optimal stability is crucial for viral replication. Journal of virology. 2002;76(11):5667-77. Epub 2002/05/07.
31. Masroori N, Cherry P, Merindol N, Li JX, Dufour C, Poulain L, et al. Gene Knockout Shows That PML (TRIM19) Does Not Restrict the Early Stages of HIV-1 Infection in Human Cell Lines. mSphere. 2017;2(3). Epub 2017/06/29.
32. Pertel T, Hausmann S, Morger D, Zuger S, Guerra J, Lascano J, et al. TRIM5 is an innate immune sensor for the retrovirus capsid lattice. Nature. 2011;472(7343):361-5. Epub 2011/04/23.
33. Goujon C, Moncorge O, Bauby H, Doyle T, Ward CC, Schaller T, et al. Human MX2 is an interferon-induced post-entry inhibitor of HIV-1 infection. Nature. 2013;502(7472):559-62. Epub 2013/09/21.
34. Jung U, Urak K, Veillette M, Nepveu-Traversy ME, Pham QT, Hamel S, et al. Preclinical Assessment of Mutant Human TRIM5alpha as an Anti-HIV-1 Transgene. Human gene therapy. 2015;26(10):664-79. Epub 2015/06/17.
