# Supplementary figures and images for: HIV-1 capsids from B27/B57+ elite controllers escape Mx2 but are targeted by TRIM5α, leading to the induction of an antiviral state
Source: PLoS Pathog. 2018 Nov 12;14(11):e1007398. doi: 10.1371/journal.ppat.1007398 (PMC6258467; doi:10.1371/journal.ppat.1007398)

Supplementary Figure 1

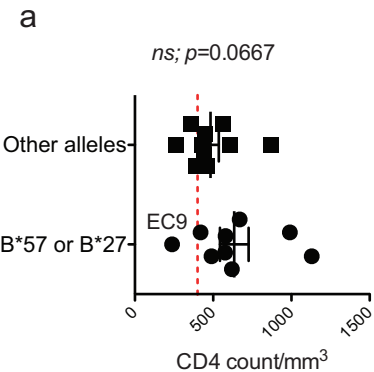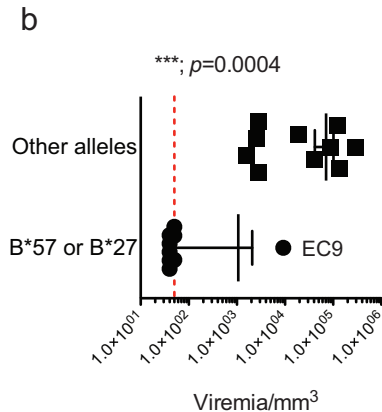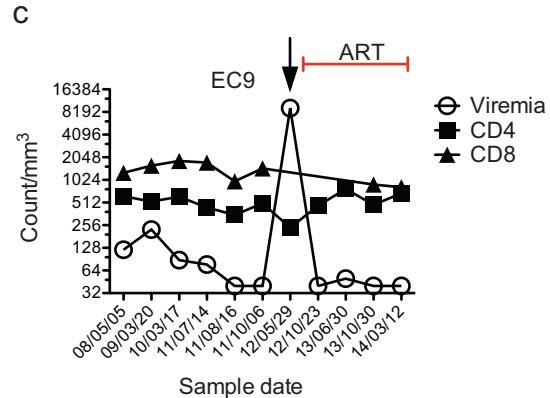

Supplement: S1 Fig — (a) CD4+ T cell counts, (b) viremia, at the time-points used in this study and according to their HLA type. B27 or B57 are grouped together. (c) Evolution of CD4, CD8 and virus counts for EC9. The black arrow indicates the time-point used in this study. The red line shows initiation and continuation of antiretroviral therapy. (PDF) [file ppat.1007398.s008.pdf]

## Supplementary Figure 2

a

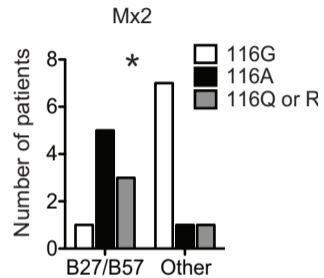

b

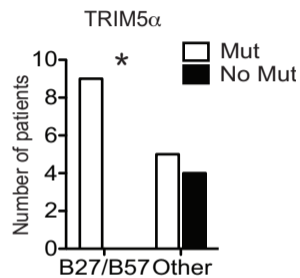

Supplement: S2 Fig — (a) Bar graph of the contingency table of mutations at the G116 position (Mx2) in individuals bearing B27/B57 or other alleles (Chi-square test; p = 0.0169). (b) Bar graphs showing the presence or the absence of mutations previously shown to be associated with TRIM5α sensitivity according to the HLA status (Fisher’s exact test; p = 0.0412). (PDF) [file ppat.1007398.s009.pdf]

# Supplementary Figure 3

**a**

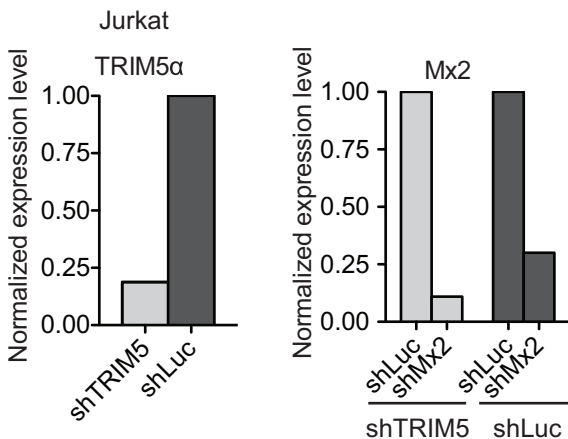

**b**

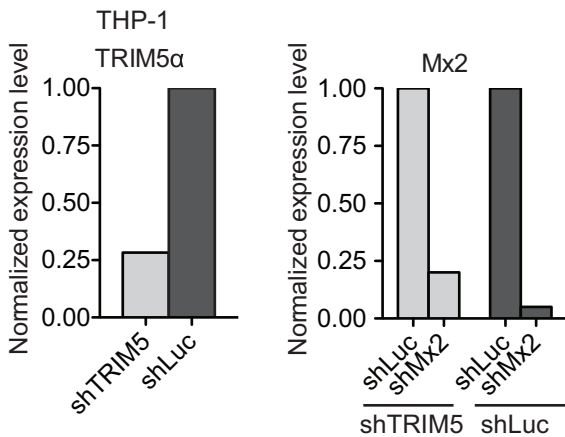

Supplement: S3 Fig — (a) mRNA levels were determined by RT-qPCR and normalized on GAPDH mRNA levels. Shown are mean mRNA levels calculated by RT-qPCR performed in duplicates on total RNA extracted from IFN-β-treated Jurkat cells, and normalized to the shLuc control. (b) Same analysis in THP-1 cells. (PDF) [file ppat.1007398.s010.pdf]

Supplementary Figure 4

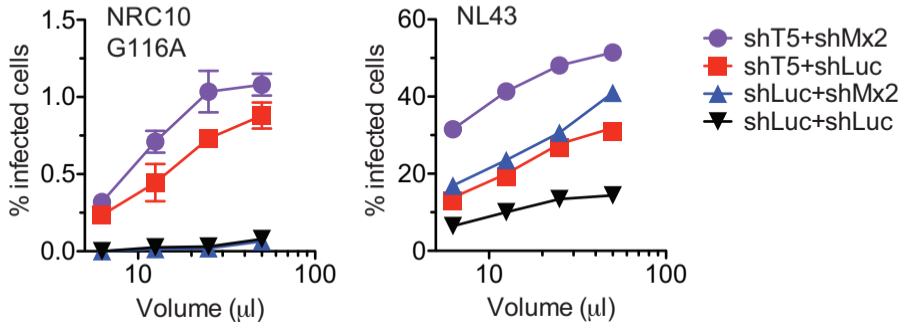

Supplement: S4 Fig — Jurkat cells knocked down for Mx2 or TRIM5α or both were infected with increasing amounts of the two HIV-1vectors. Infectivity was measured by FACS as the % of GFP+ cells 48 h post-infection. (PDF) [file ppat.1007398.s011.pdf]

Supplementary Figure 6

a

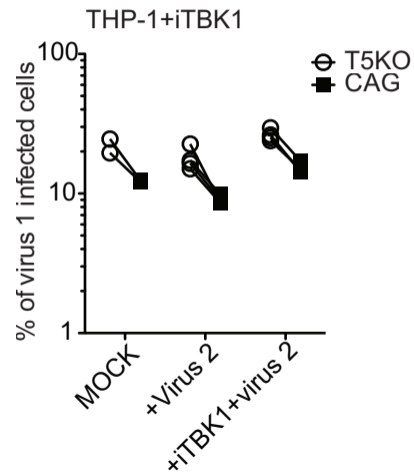

b

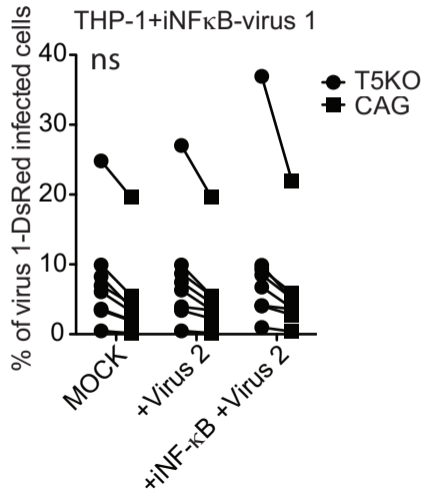

c

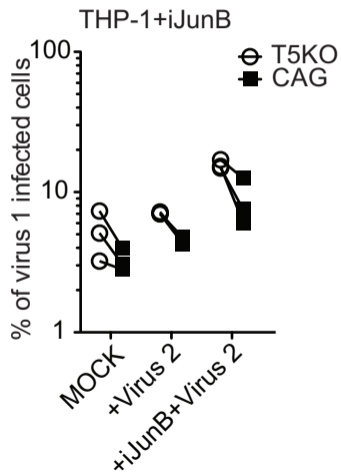

Supplement: S6 Fig — THP-1 cells were pre-treated or not with (a) BX795 (iTBK1), (b) BAY11-7085 (iNF-κB) or (c) SP600125 (iAP-1) for 1 h, infected with DsRed-expressing chimeric vectors (“virus 1”), and 48 h later infected with NRC1GFP (“virus 2”). Infectivity of DsRed-virus 1 was assessed by flow cytometry 48 h later. Data are from the same infections as those shown in Fig 5C, Fig 6D and Fig 6E, respectively. (PDF) [file ppat.1007398.s013.pdf]

Supplementary Figure 7

**a**

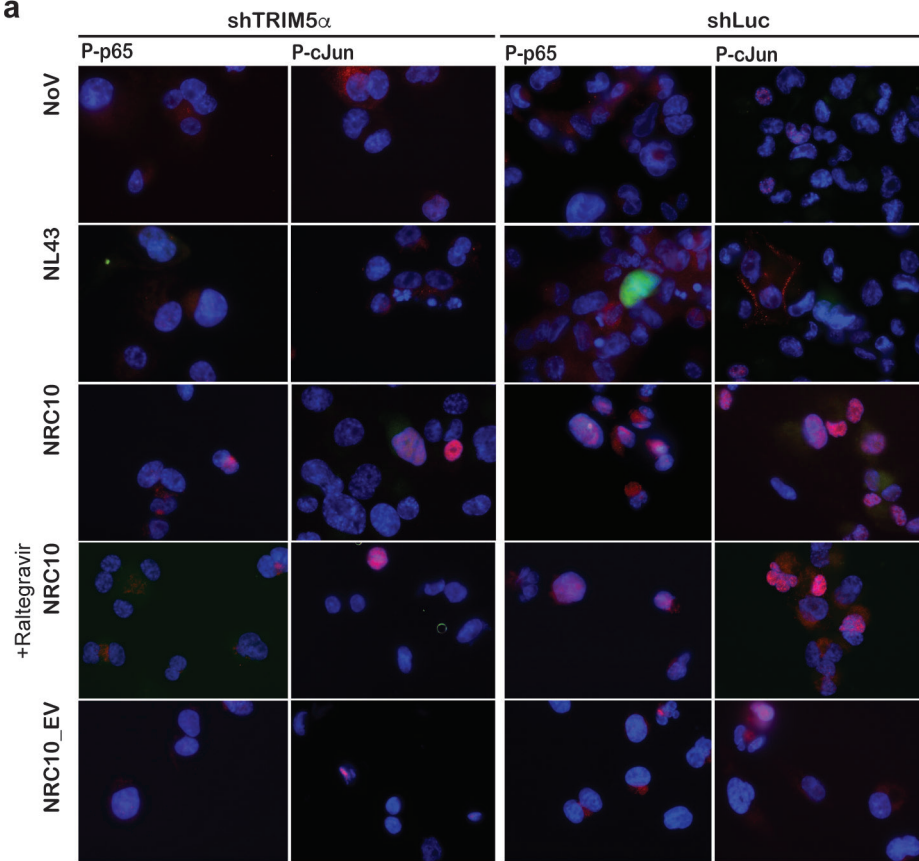

**b**

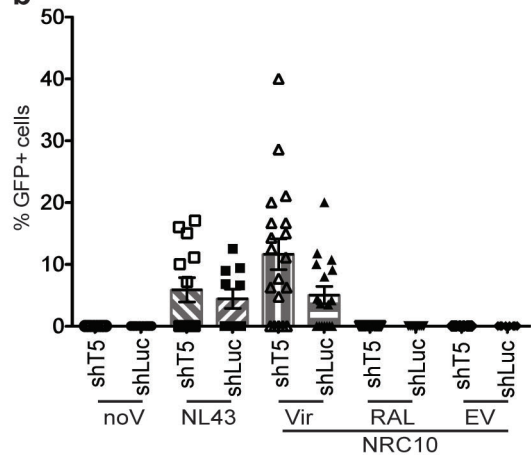

**c**

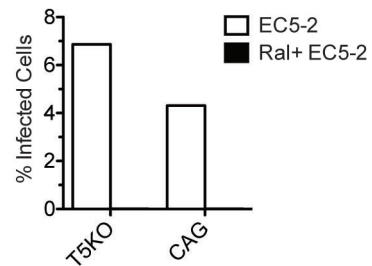

Supplement: S7 Fig — (a) Microscopy images corresponding to Fig 6A with the GFP field included. (b) Frequency of infected (GFP+) cells quantified by analyzing ≥100 cells from ≥ 10 pictures and plotted according to TRIM5α expression and viral infection. The Kruskal-Wallis test and the Dunn's Multiple Comparison Test were used to assess statistical significance. Shown are means with SEM. noV = No virus, Vir = virus, RAL = Raltegravir, EV = empty vector. (c) T5KO and control THP-1 cells were treated for 60 min with Raltegravir (RAL) or left untreated then infected with the GFP-expressing EC5-2 vector at a CRFK MOI = 2. Infectivity (% GFP+ cells) was measured by FACS at 48 h post-infection. (PDF) [file ppat.1007398.s014.pdf]

Supplementary Figure 8

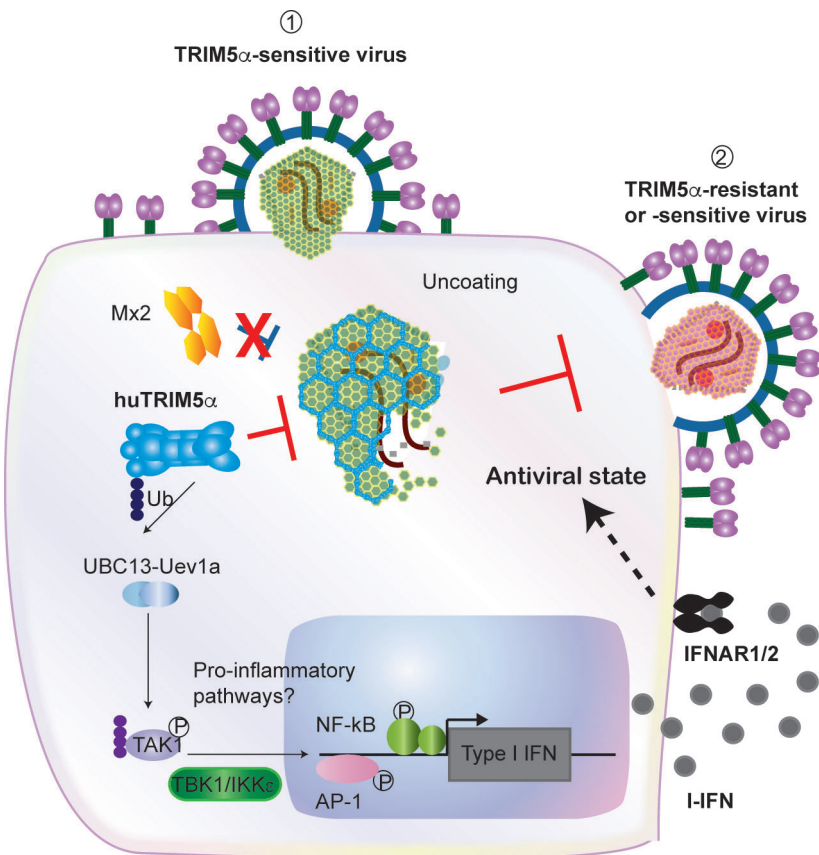

Supplement: S8 Fig — Following entry, viruses from a B27/B57+ subject escape Mx2 restriction but are recognized by TRIM5α. TRIM5α disrupts the proper uncoating process and may trigger pro-inflammatory signals through Ubc13- and TAK1-dependent signaling. In THP-1 cells, this leads to activation of NF-κB and AP-1 and production of type I IFN production that signals through IFNAR1/2 to induce an antiviral state that blocks infection from TRIM5α-sensitive as well as TRIM5α-resistant HIV-1 viruses. In Jurkat cells, the antiviral state is induced in an IFN-I-independent manner. (PDF) [file ppat.1007398.s015.pdf]
