# Supplementary material for: HIV-1 capsids from B27/B57+ elite controllers escape Mx2 but are targeted by TRIM5α, leading to the induction of an antiviral state
Source: PLoS Pathog. 2018 Nov 12;14(11):e1007398. doi: 10.1371/journal.ppat.1007398 (PMC6258467; doi:10.1371/journal.ppat.1007398)

Supplementary Figure 5

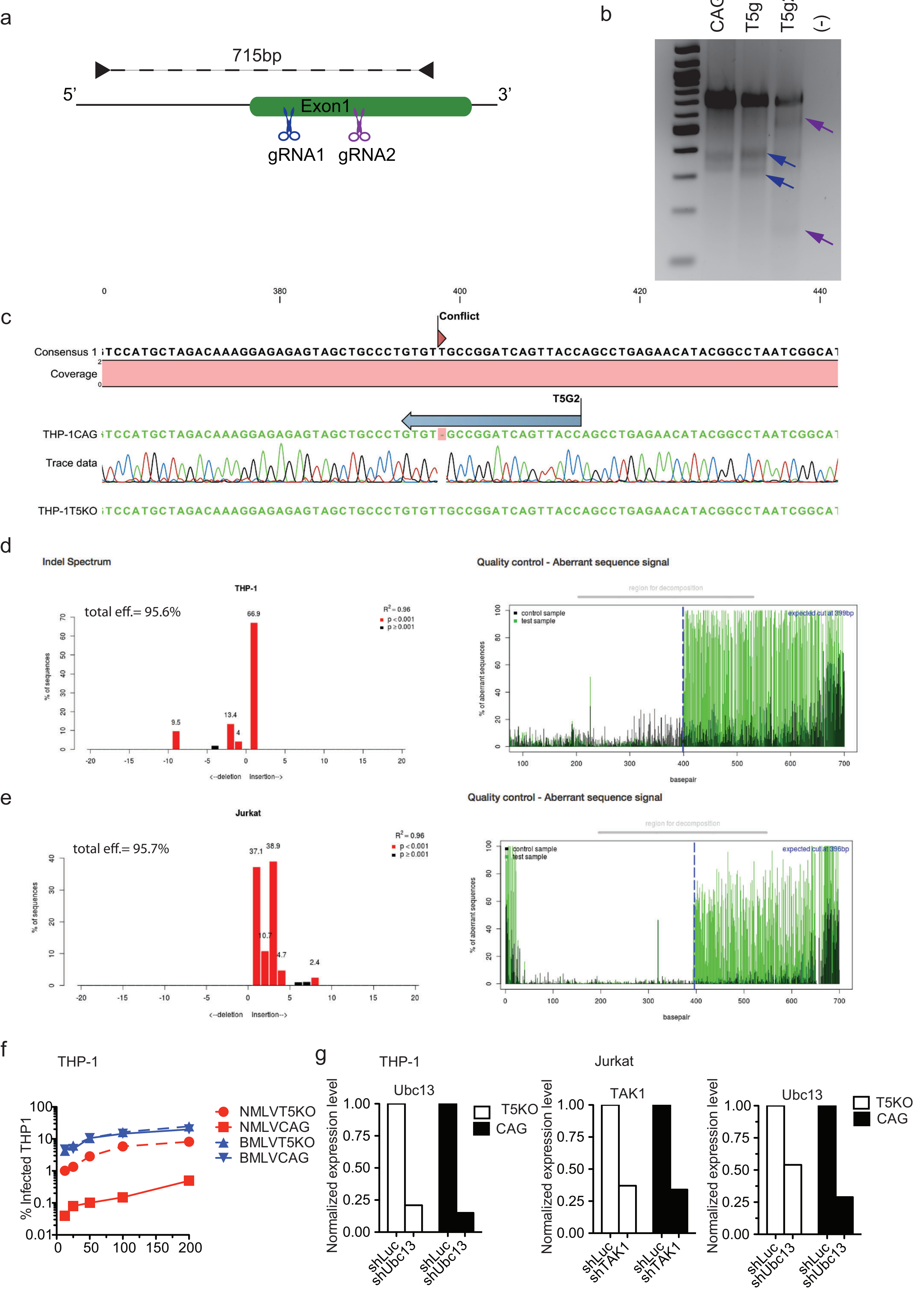

Supplement: S5 Fig — (a) Cas9 was targeted to exon 1 of the TRIM5 gene (green) by two selected gRNAs, whose binding sites are schematized with scissors. Arrowheads indicate the positions of the binding sites for the ODNs used in the PCR-Surveyor assay. (b) Surveyor assay. Briefly, PCR products amplified from 293T cells transfected with pLCv2-hT5g1, pLCv2-hT5g2, or pLCv2-CAG (control) were subjected to denaturation, reannealing, and digestion with the Surveyor enzyme. Arrows indicate cleavage products of the expected size. (c) Sanger sequencing analysis. THP-1 cells were transduced with lentiviral vectors produced using pLCv2-hT5g2 or the control vector, pLCv2-CAG. Following puromycin selection, the targeted TRIM5 locus was PCR amplified and the PCR product was Sanger sequenced. The figure shows an alignment of the obtained sequence plots. (d, e) Decomposition of sequencing plots by TIDE assay. The graphs on the left show the percentages of aberrant peaks upstream and downstream of the cut site in the sequencing reactions shown in panel c in THP-1 (d) and in Jurkat (e) cells. The graphs on the right display the frequency of aberrant sequence signals in TRIM5 sequences in corresponding T5KO (test sequences in green) and control (CAG in black) cells. The percentage of indel-containing alleles was computed by the TIDE assay. (f) T5KO and control THP-1 were infected with N-MLVGFP and B-MLVGFP. Infectivity was assessed by flow cytometry 72 h post-infection. (g) Knockdown validation for Ubc13 and TAK1 in TRIM5α knockout and control cells. mRNA levels were determined by RT-qPCR and normalized on GAPDH mRNA levels. Shown are mean mRNA levels calculated by RT-qPCR performed in duplicates on total RNA extracted from TRIM5-KO and control CAG THP-1 and Jurkat cells as indicated. (PDF) [file ppat.1007398.s012.pdf]
